# Supplementary material for: How EEG preprocessing shapes decoding performance
Source: Commun Biol. 2025 Jul 10;8:1039. doi: 10.1038/s42003-025-08464-3 (PMC12246244; doi:10.1038/s42003-025-08464-3)
Supplement: Supplementary file 2 — Supplementary Information [file 42003_2025_8464_MOESM2_ESM.pdf]

Supplementary Materials for

How EEG preprocessing shapes decoding performance

Roman Kessler *et al.*

*Corresponding author. Email: rkesslerx@gmail.com

This PDF file includes:

Supplementary Text
- Variability due to random seed in autoreject
- Influence of latent leakage on decoding performance
- Alternative order of preprocessing steps
- Influence of the participant in the LMMs

Figures S1 to S34
Tables S1 to S9

Supplementary Text

Variability due to random seed in autoreject

The *autoreject* package[1,2] shows considerable variability in the number of dropped epochs, when applied to the same data but using a different random seed. To illustrate this, we used in a first analysis one example participant in the ERN experiment to show the influences of the random seed in the autoreject function, and a random seed in trial sampling prior to fitting the autoreject model. Some variability due to the seed in sampling the epochs for fitting autoreject is expected. We therefore tested 19 random seeds at both stages. Figure S29 illustrates, that substantial variability is introduced by random seeds at both stages. However, figure S30 illustrates, that similar epochs are rejected for different seeds, i.e., if a seed rejects more epochs, it usually contains epochs also rejected using a different seed.

In a second analysis, using all participants, we tested the intraclass correlation coefficient[3] (ICC, version 1,1) to illustrate the reliability of accuracy values across different random seeds using the Python package *pingouin* (v. 0.5.5). We used the N170 experiment and randomly selected three example pipelines (1. ocular ICA, muscle ICA, 45 Hz LPF, 0.5 Hz HPF, average reference, linear detrending, 200 ms baseline, 2. no ICAs, 45 Hz LPF, 0.5 Hz HPF, average reference, linear detrending, 200 ms baseline, and 3. no ICAs, 45 Hz LPF, 0.1 Hz HPF, average reference, linear detrending, no baseline) without autoreject, and added an autoreject step in the *interpolate* version and in the *reject* version, resulting in 6 forking paths. For each forking path, we applied autoreject with 5 different seeds set in the autoreject estimation (seeds 0, 1, 2, 3, 4) while keeping the sampling seed constant (seed 0). Further, we kept the seed in autoreject constant (seed 0), and varied the sampling seed (seeds 0, 1, 2, 3, 4). The resulting data were used for decoding with EEGNet. This was repeated for all 40 participants.

We computed ICCs separately across different seed numbers and participants, for each pipeline and for all seed types (autoreject seed or sampling seed). All resulting ICCs were greater than 0.867 (all $p < 0.05$, false discovery rate-corrected), indicating excellent agreement[4] between different seeds when evaluating participant test accuracies.

Lastly, we calculated the ICCs of the average accuracies (across participants) for each forking path, with the seeds as raters. All ICCs were greater than 0.466 (all $p < 0.05$, false discovery rate-corrected), indicating fair agreement[4] between different seeds when evaluating the test accuracies across forking paths.

Influence of latent leakage on decoding performance

To investigate latent leakage caused by joint preprocessing of the data later used in the different cross-validation splits, we analyzed the N170 dataset including all participants. We investigated ocular ICA, autoreject, and HPF in separate analyses, by choosing a simple preprocessing pipeline for each step (i.e., corresponding to one forking path). We sealed the respective preprocessing step by meticulously separating train and test sets. We decoded from the leaky and the sealed processing pipelines and compared decoding performances.

HPF: For the leaky pipeline, we filtered the participants entire raw time series in one step using a HPF cutoff of 0.1 Hz, corresponding to a filter length of 33 s (table S6). We divided the raw time series into 5 segments of equal length and extracted epochs from each segment (baseline correction of 200 ms, no detrending). We used Cz as reference. No other artifact correction steps were performed. We trained and tested an EEGNet classifier similarly to before, using a 5-fold cross-validation with balanced accuracy. For the sealed pipeline, we divided the raw time series into 5 segments prior to applying the HPFs on each segment individually. The remaining steps were the same.

ICA: For both pipelines, we first applied a HPF of 0.5 Hz, and a LPF of 45 Hz. For the leaky pipeline, we estimated the ICA, detected and rejected the artifactual components on the entire time series. The time series was divided into 5 segments, epochs were extracted from each segment as before, and these were used in the separate cross-validation folds. For the sealed pipeline, we split already the filtered time series into 5 segments, and fitted the ICA only on the 4 (out of 5) segments that were later used for training. We applied the ICA to the train and test segments. This effectively excluded the test segment from the estimation of the ICA and the rejection thresholds. We repeated this for all 5 folds, extracted the epochs, and used them for the respective training and testing. Balanced accuracies were averaged across folds. To ensure an equal number of trials across the leaky and sealed versions of a pipeline, we always discarded trials near the segment boundaries.

Autoreject: For both pipelines, we first deployed HPFs and LPFs as before. Then we epoched the data as before. We used the "interpolate" version of autoreject (see Methods). For the leaky pipeline, we estimated the autoreject thresholds using all epochs. The epochs were then divided into 5 parts to be used in cross-validation. For the sealed version, we divided the epochs before the autoreject step. For each fold of the cross-validation, the autoreject parameters were estimated on the 4 segments in the train set, and the rejection threshold was applied to train and test sets. Balanced accuracies were averaged across folds.

We used a one-tailed, paired sample *T*-test for each preprocessing step to evaluate, whether the leaky pipeline resulted in higher decoding accuracies than the sealed pipeline. Figure S32 illustrates, that there was no higher decoding accuracies for leaky pipelines as compared to sealed pipelines (all $p > 0.638$).

Alternative order of preprocessing steps

We tested one alternative order of preprocessing steps in a reduced multiverse. There we applied the steps in the order of (1) re-referencing, (2) HPF & LPF, (3) ocular artifact correction, (4) muscle artifact correction, (5) baseline correction & detrending, and (6) autoreject. In this version, we always performed a baseline correction (either 200 ms or 400 ms). For detrending, we chose between offset (subtract mean) and linear (apply linear function and keep residuals). We either used autoreject in the interpolate version, or did not apply it. The remaining choices were the same. Following a similar intuition than with the main multiverse, we also filtered the data with 1 Hz prior to ICA[5].

Figure S26 illustrates the decoding performances (accuracies and *T*-sums) for classifiers trained on data preprocessed with the alternative multiverse order. The magnitudes were comparable to those reported in our main multiverse pipeline (Fig. 3). We also computed the corresponding marginal means (fig. S27). Most effects were in a similar direction as in our main multiverse results (Fig. 5). However, the LPF in the EEGNet framework had quite opposite effects. This can be explained by the fact that it is followed by the muscle artifact correction step, which had no effect (i.e., did not drop any components) when a LPF of 6 Hz or 20 Hz was applied. This, in turn, resulted in all forking paths with these filter settings having no muscle artifacts removed, which in turn were predictive and increased decoding performance.

<u>Influence of the participant in the LMMs</u>

Within the LMMs constructed to explain the accuracies of trained EEGNet models, we included random intercepts for the 40 participants. Figure S22 illustrates, how the distribution of age, sex, handedness corresponds to the random intercept, i.e., to the individual participants offset in decoding accuracy. Age and handedness did not show any noticeable pattern. We further tested the difference of random intercepts between sexes using a Mann–Whitney *U* test with Benjamini Hochberg false discovery rate adjustment[6] for 7 experiments (fig. S22, middle column). No significant differences were observed. We repeated the same analysis for time-resolved LMM results, which were fitted with the averaged decoding accuracies over time as dependent variables (fig. S23). No significant differences were observed.

In addition, figure S24 illustrates the estimated random intercepts for each participant across the 7 experiments. One might hypothesize that participants with a high random intercept in one experiment would also have a high random intercept in the other experiments, indicating that it is easier to decode of EEG signals from some participants, but not others. For each pair of experiments, we computed Pearson correlation coefficients between the random intercepts of the same participants, and corrected for the false discovery rate using Benjamini Hochberg procedure. Figure S24 generally shows no such a relationship. The only significant correlations were observed between the random intercepts of LRP and N170 (fig. S24)[7]. We repeated the same analysis for time-resolved LMM results, which were fitted with the individual-participant averaged decoding accuracies over time as dependent variables instead of group T-sum values (fig. S25). No significant correlations were observed.

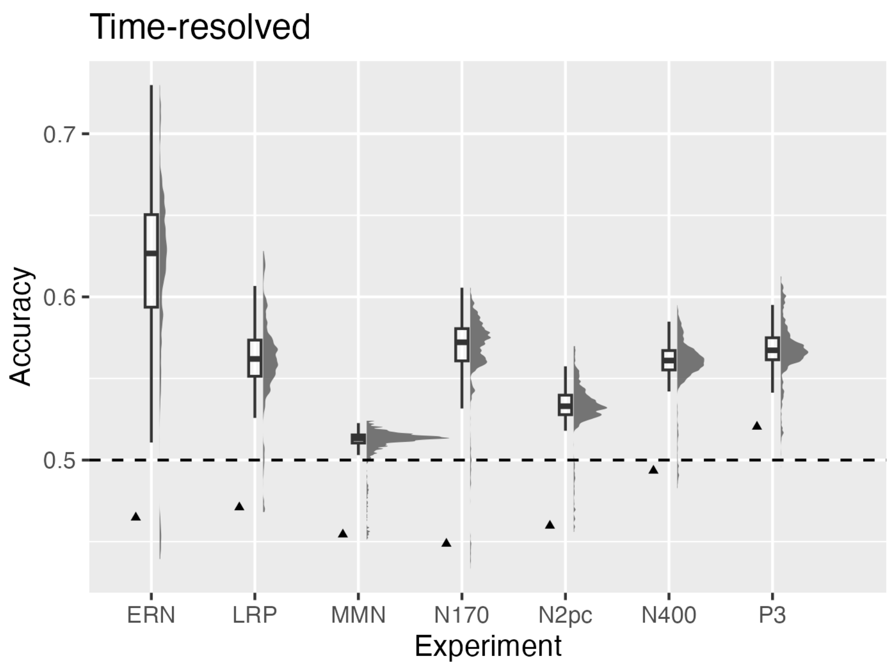

Fig. S1. Overview of average time-resolved decoding accuracies.
Average post-baseline decoding accuracies (y-axis) are plotted for each forking path, further averaged across participants, separately for each experiment (x-axis). Unlike Figure 3B, it is the average decoding accuracy that is plotted, not the *T*-sum. Triangles indicate the forking path without any preprocessing. Boxes represent the interquartile range (25th to 75th percentile), with the median indicated by a solid black line. Whiskers extend to the most extreme values within 1.5 times the interquartile range from the lower and upper quartiles.

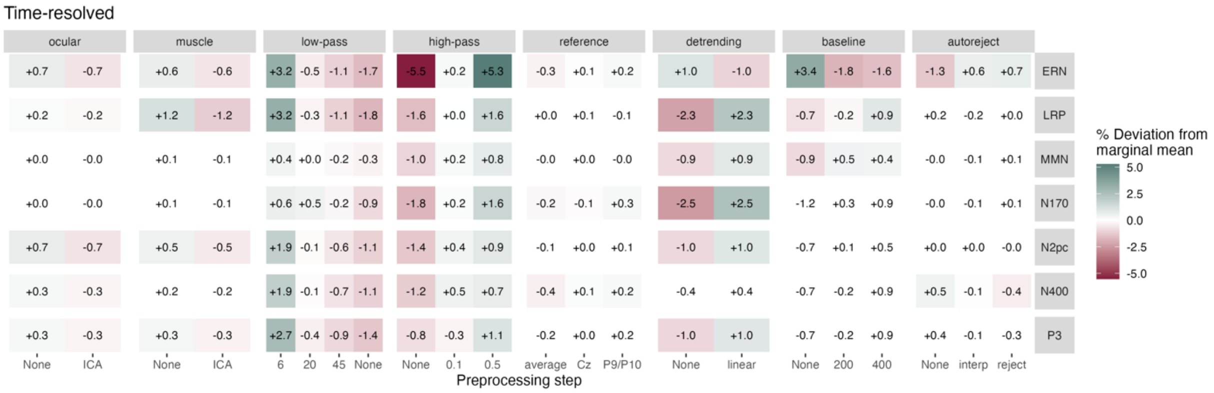

Fig. S2. Influence of preprocessing steps on time-resolved decoding performance. The average post-baseline decoding accuracy is used as a proxy for decoding performance instead of T-sum. Only cells with significant F-test ($p < 0.05$) are colored. See Figure 5 for more details.

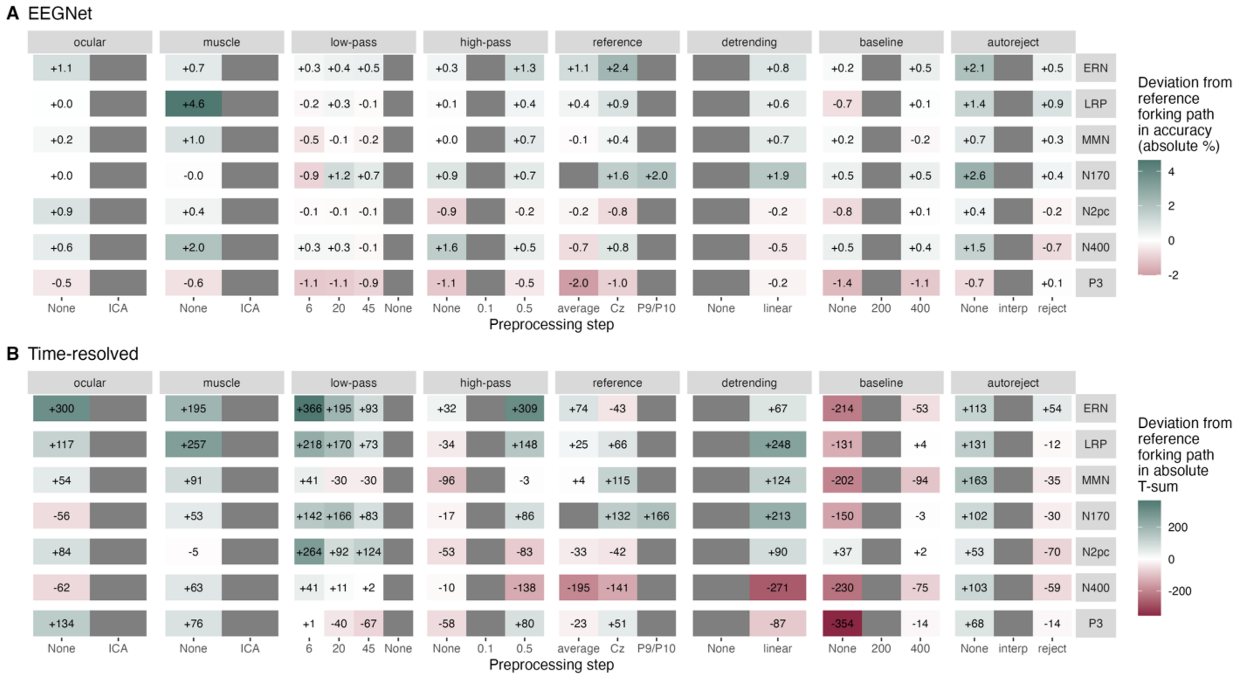

Fig. S3. Influence of varying one single preprocessing steps on decoding performance. Absolute increase or decrease in decoding performance – either accuracy (EEGNet, **A**) or *T*-sum (time-resolved, **B**) – is depicted within each tile. Change in decoding performance for each level (x-axis) of preprocessing step (horizontal panels) are illustrated in comparison to the example forking path (asterisks in Fig. 1) for each respective experiment (vertical panels). Grey tiles indicate steps of the respective reference forking path. Color scales differ in **A** and **B**. *Ocular*: ocular artifact correction; *muscle*: muscle artifact correction; *ICA*: independent component analysis, *low-pass*: low-pass filter in Hertz; *high-pass*: high-pass filter in Hertz; *baseline*: baseline interval in milliseconds; *autoreject* version either interpolate (*interp*) or reject artifact-contaminated trials (*reject*).

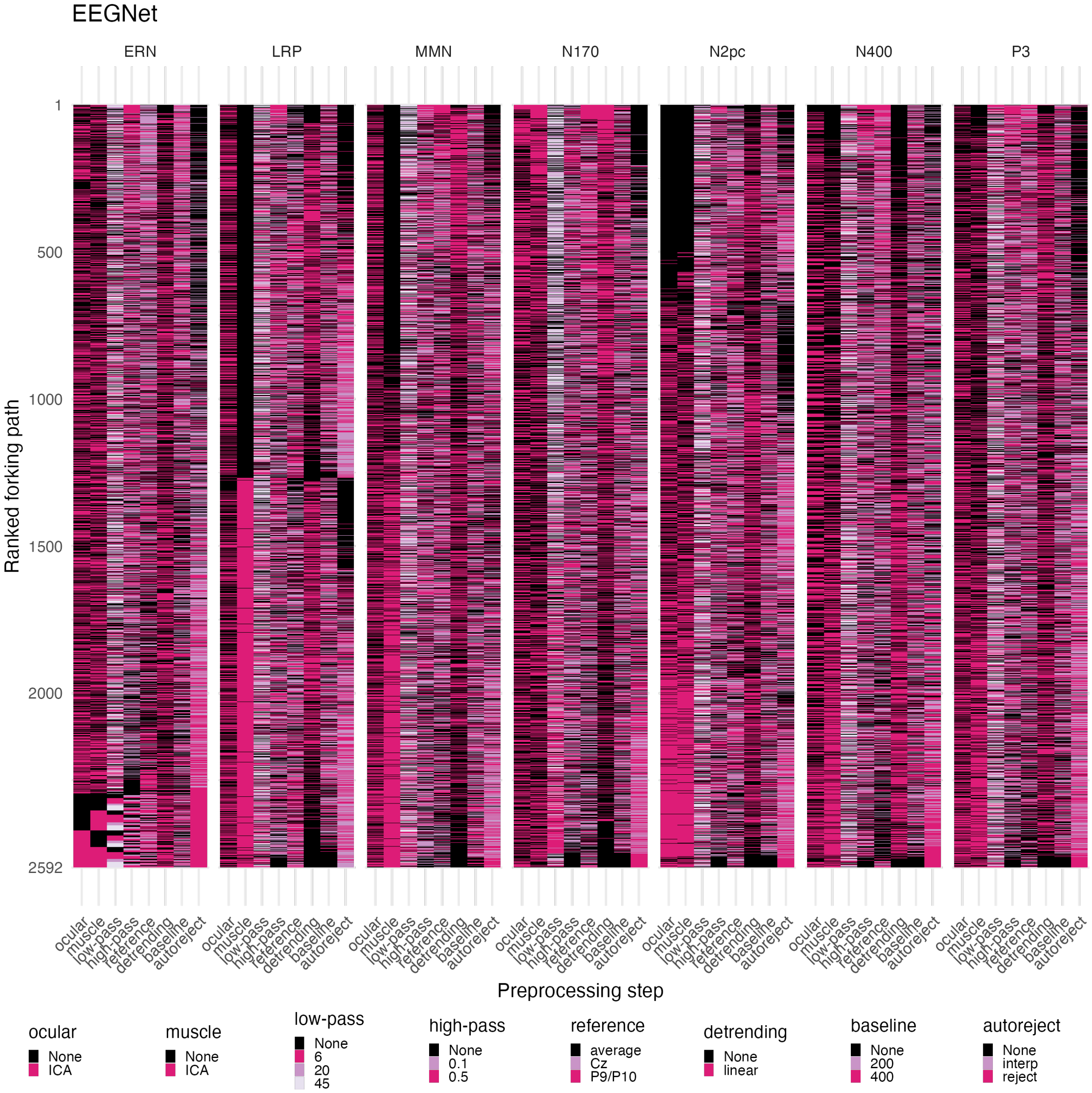

Fig. S4. Ranked forking path performances (EEGNet decoding).
For each experiment (horizontal panels), the forking paths were ranked from highest (#1) to lowest (#2592) decoding performance. The individual variations of each preprocessing step (x-axes) are color-coded. Each individual row corresponds to one data point in Figure 3A. *Ocular*: ocular artifact correction; *muscle*: muscle artifact correction; *ICA*: independent component analysis, *low-pass*: low-pass filter in Hertz; *high-pass*: high-pass filter in Hertz; *baseline*: baseline interval in milliseconds; *autoreject* version either interpolate (*interp*) or reject artifact-contaminated trials (*reject*).

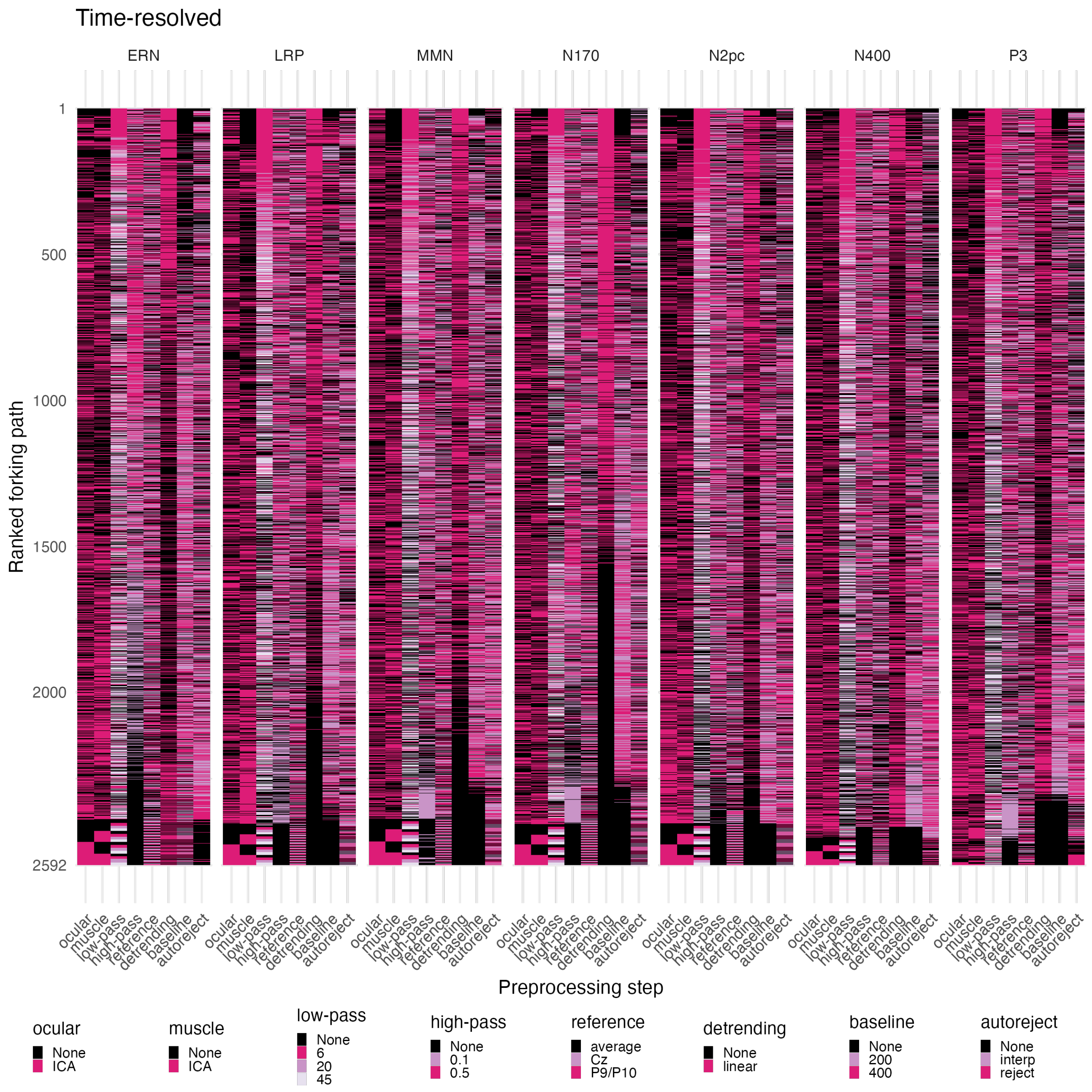

Fig. S5. Ranked forking path performances (time-resolved decoding).
For each experiment (horizontal panels), the forking paths were ranked from highest (#1) to lowest (#2592) decoding performance. The individual variations of each preprocessing step (x-axes) are color-coded. Each individual row corresponds to one data point in Figure 3B. *Ocular*: ocular artifact correction; *muscle*: muscle artifact correction; *ICA*: independent component analysis, *low-pass*: low-pass filter in Hertz; *high-pass*: high-pass filter in Hertz; *baseline*: baseline interval in milliseconds; *autoreject* version either interpolate (*interp*) or reject artifact-contaminated trials (*reject*).

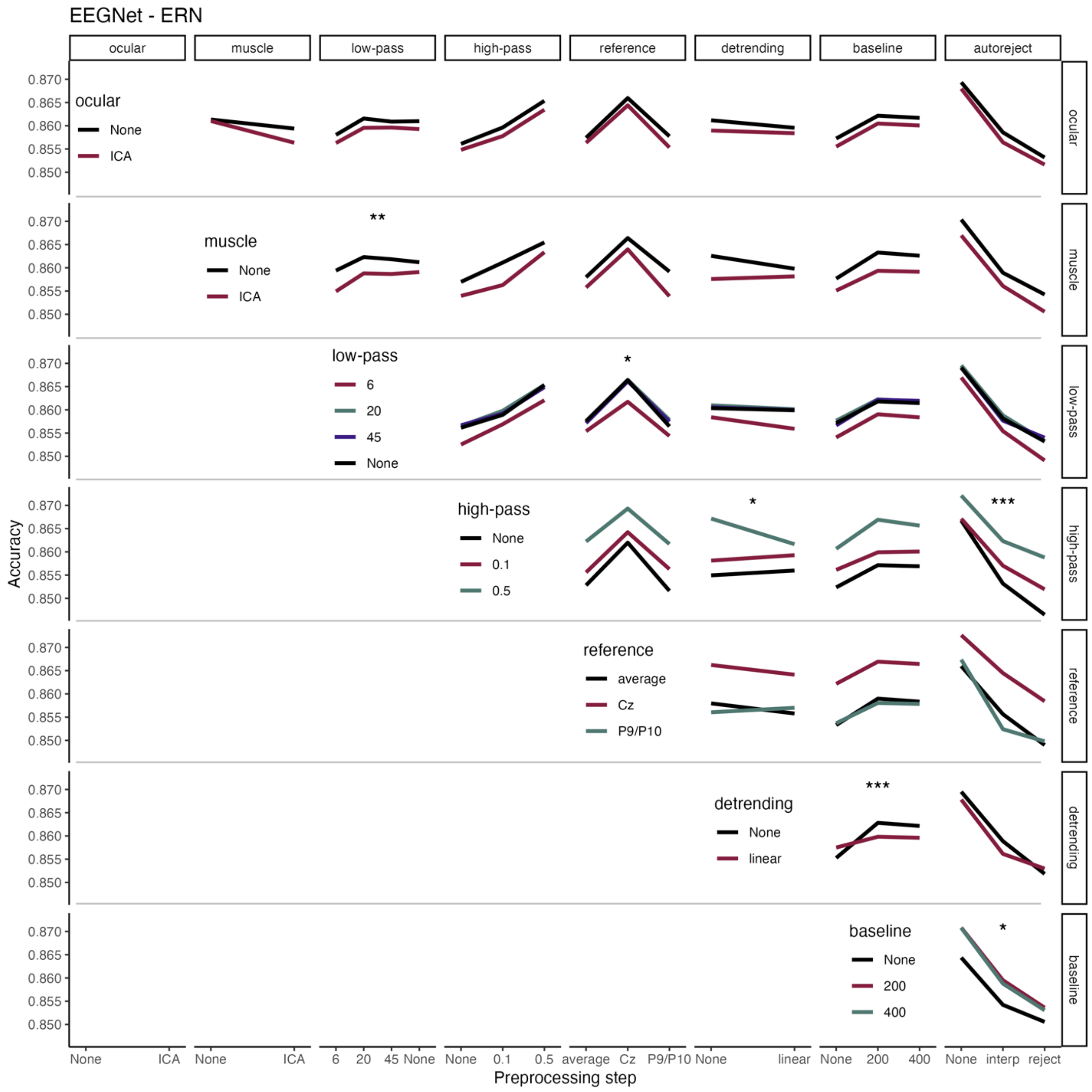

Fig. S6. Interactions between preprocessing steps on decoding performance for ERN and EEGNet decoding.

Horizontal and vertical panels illustrate the different preprocessing steps. The individual preprocessing choices are illustrated on the x-axes and color-coded. The color legends on the diagonal refer to each horizontal panel. (Balanced) Decoding accuracies are shown on the y-axes. Stars indicate the significance ('*' $p < 0.05$; '**' $p < 0.01$; '***' $p < 0.001$). *Ocular*: ocular artifact correction; *muscle*: muscle artifact correction; *ICA*: independent component analysis, *low-pass*: low-pass filter in Hertz; *high-pass*: high-pass filter in Hertz; *baseline*: baseline interval in milliseconds; *autoreject* version either interpolate (*interp*) or reject artifact-contaminated trials (*reject*).

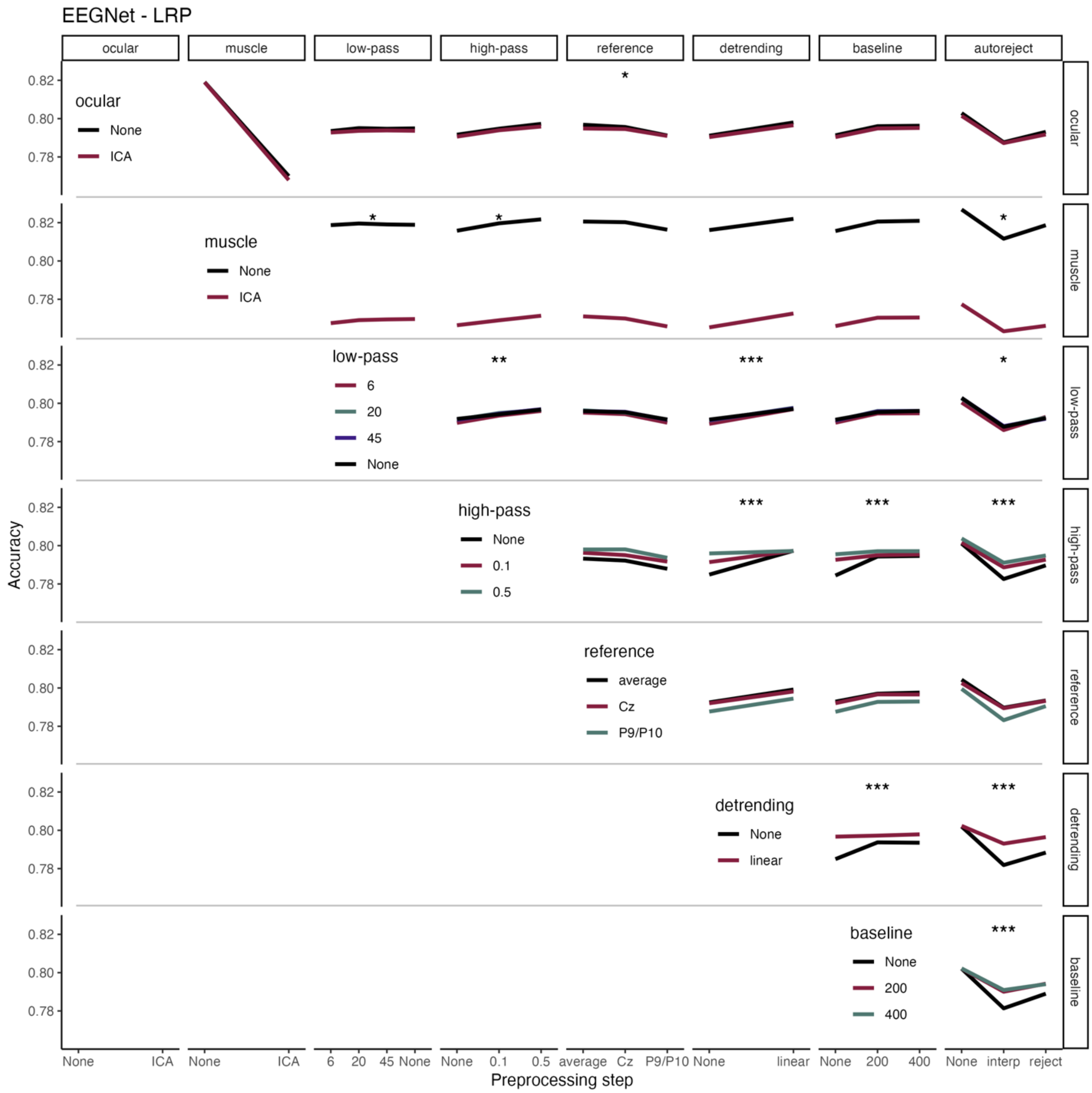

Fig. S7. Interactions between preprocessing steps on decoding performance for LRP and EEGNet decoding.
See Figure S5 for details.

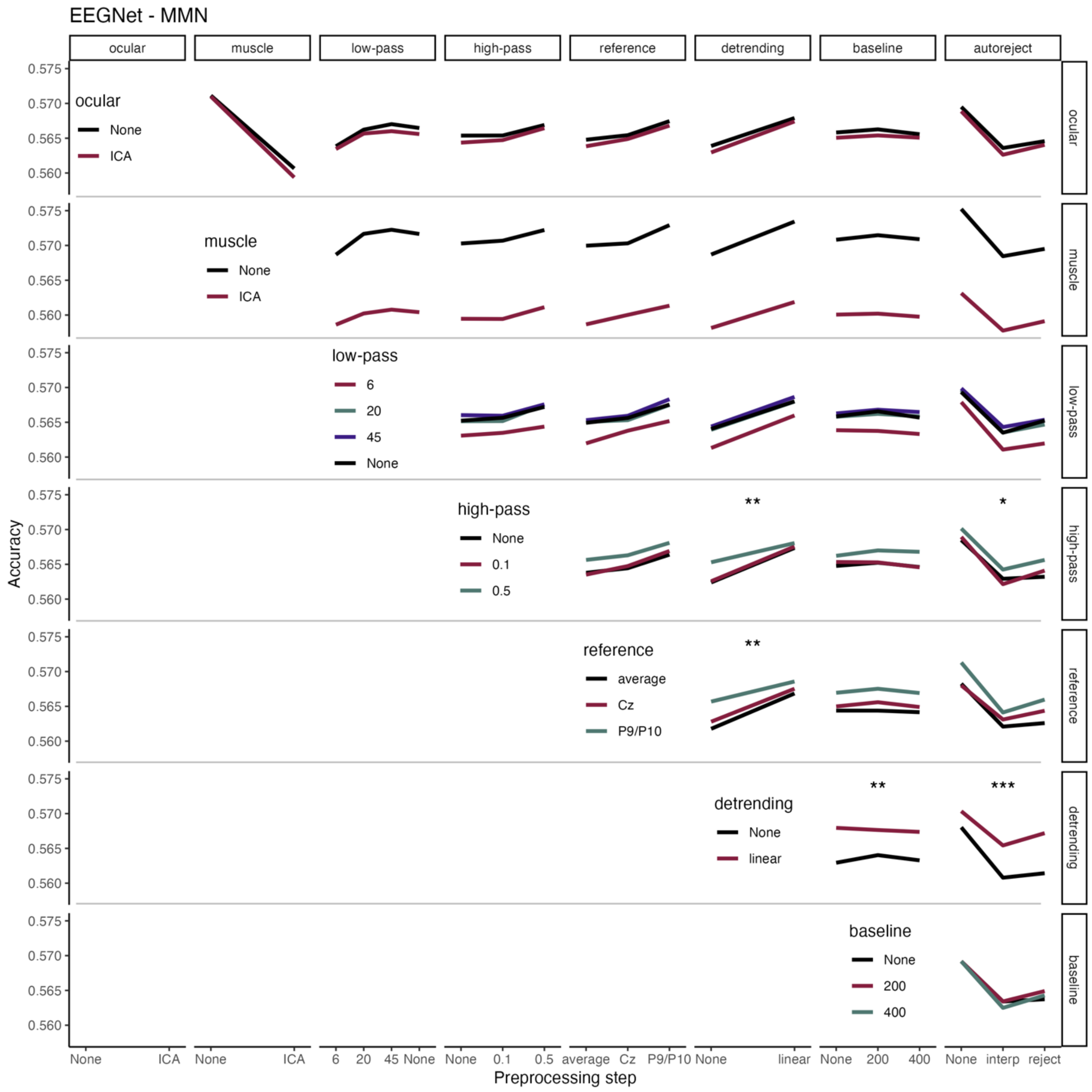

Fig. S8. Interactions between preprocessing steps on decoding performance for MMN and EEGNet decoding.

See Figure S6 for details.

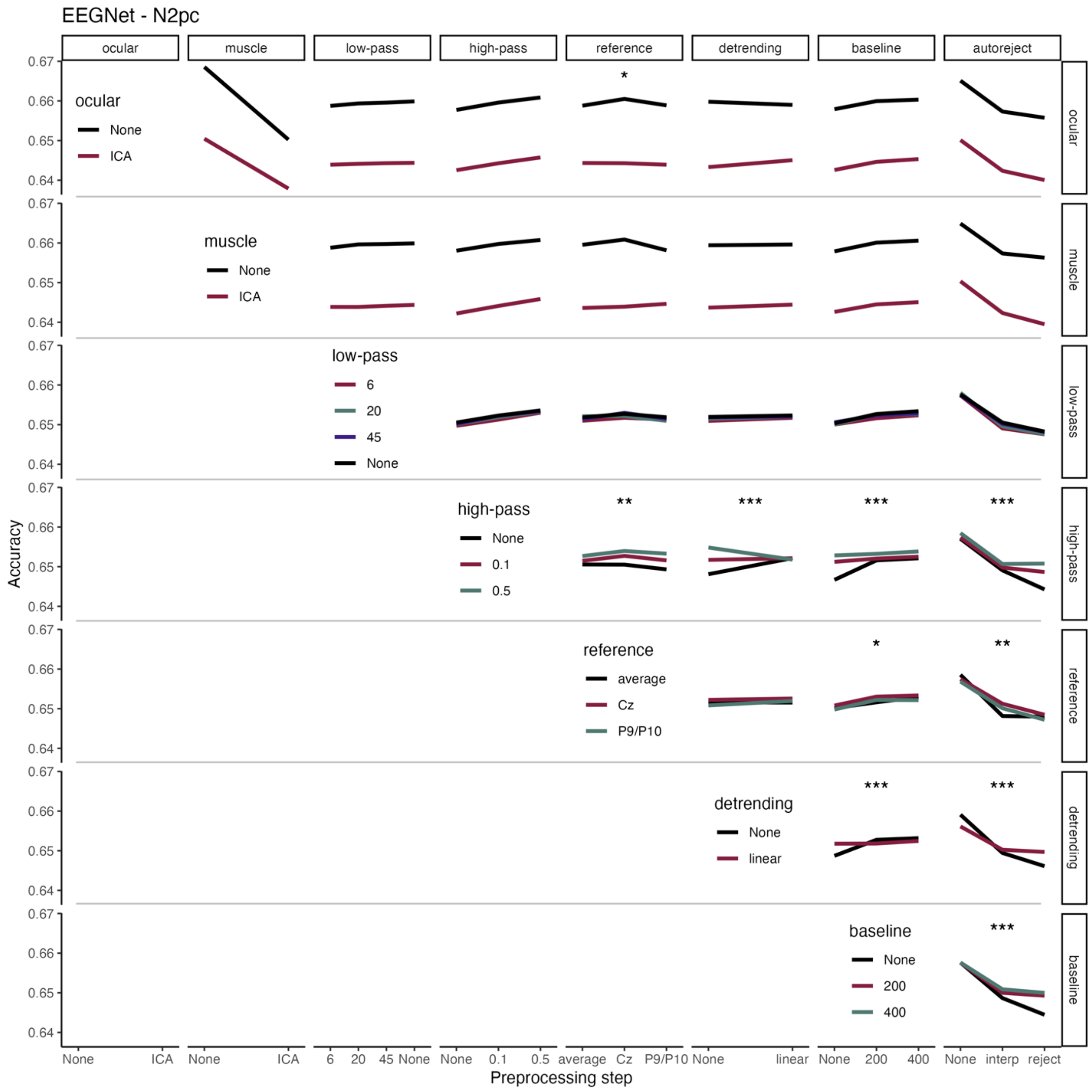

Fig. S9. Interactions between preprocessing steps on decoding performance for N2pc and EEGNet decoding.
See Figure S6 for details.

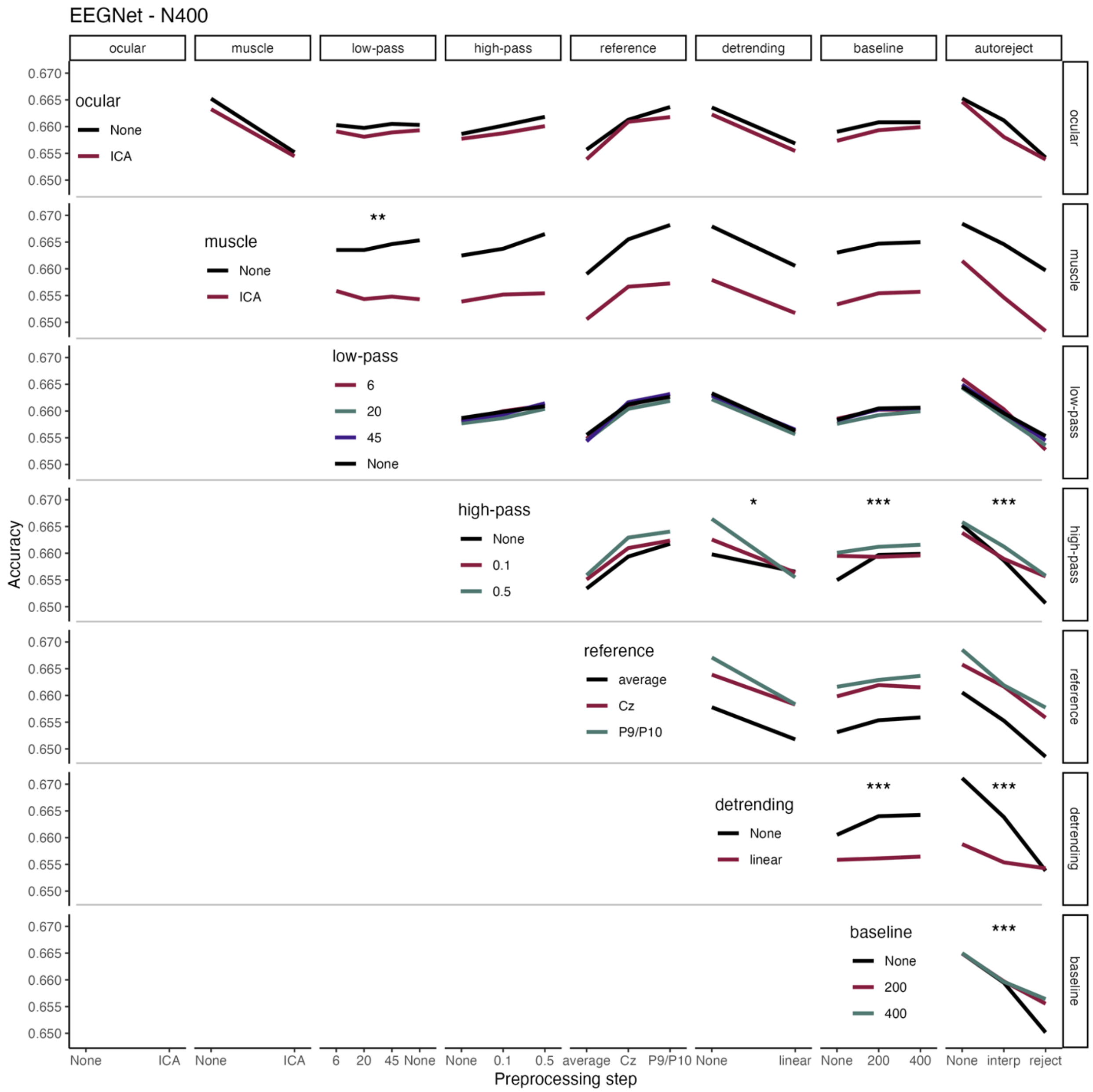

Fig. S10. Interactions between preprocessing steps on decoding performance for N400 and EEGNet decoding.
See Figure S6 for details.

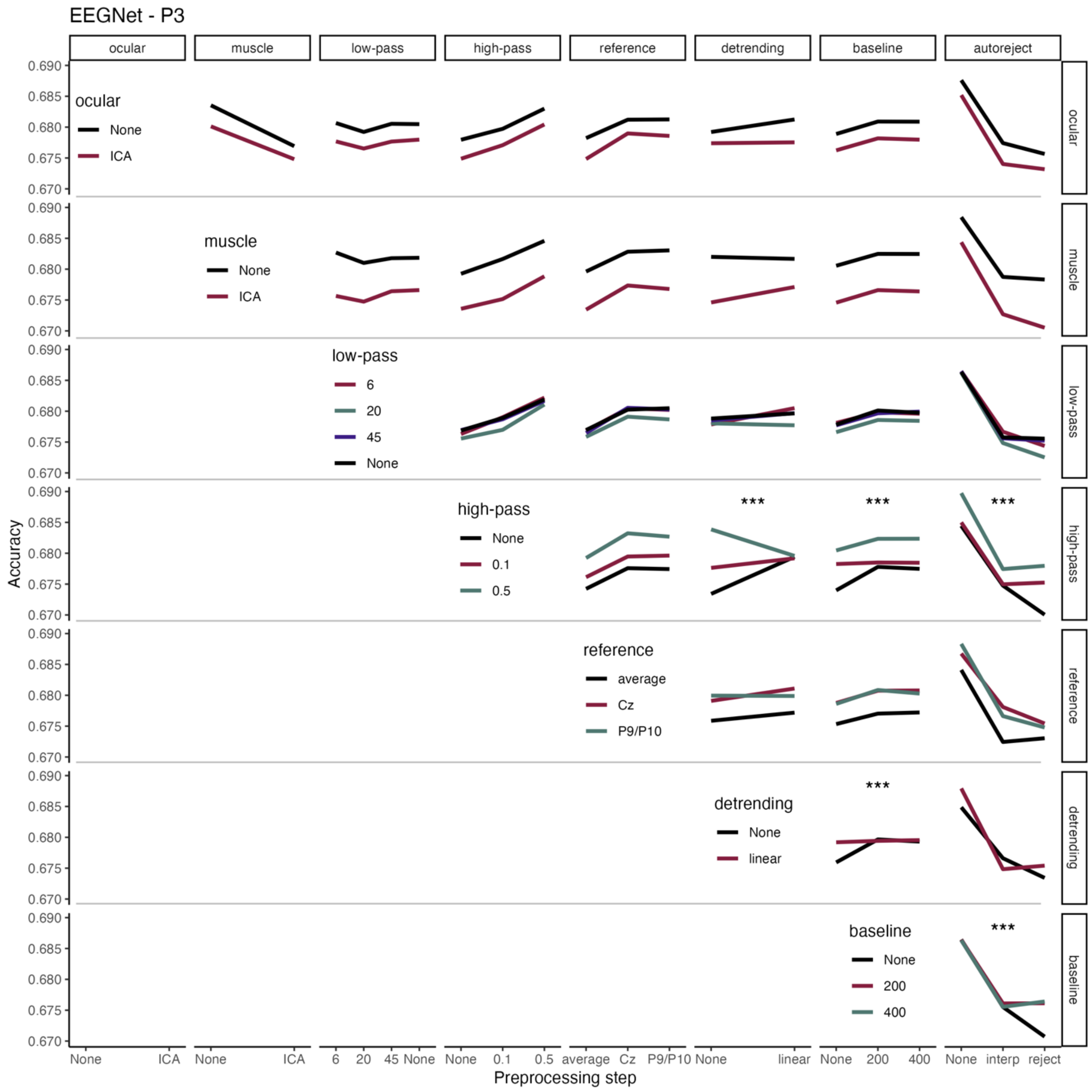

Fig. S11. Interactions between preprocessing steps on decoding performance for P3 and EEGNet decoding.
See Figure S6 for details.

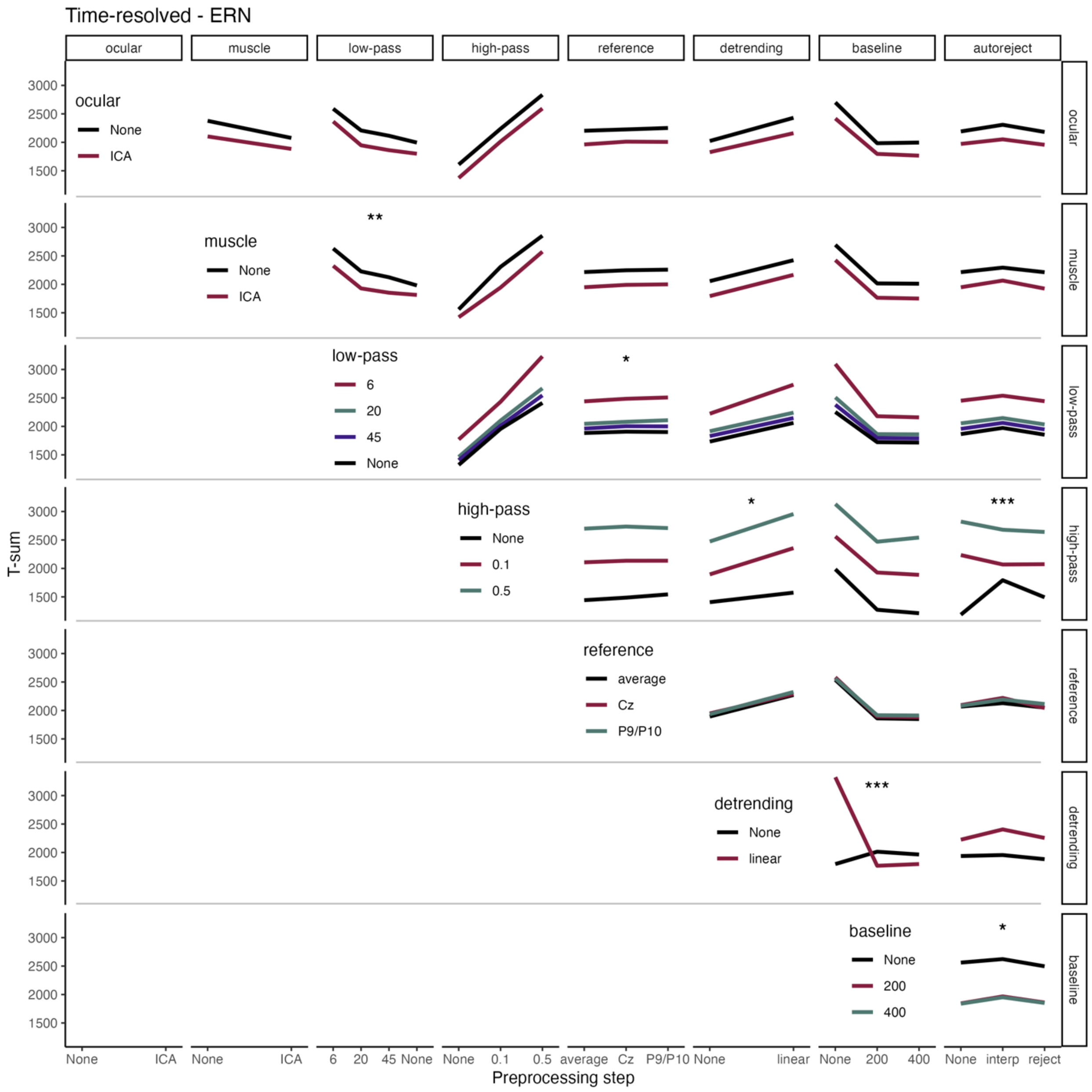

Fig. S12. Interactions between preprocessing steps on decoding performance for ERN and time-resolved decoding.
Horizontal and vertical panels illustrate the different preprocessing steps. The individual preprocessing choices are illustrated on the x-axes and color-coded. The color legends on the diagonal refer to each horizontal panel. T-sum is shown on the y-axes. Stars indicate the significance ('*' $p < 0.05$; '**' $p < 0.01$; '***' $p < 0.001$). *Ocular*: ocular artifact correction; *muscle*: muscle artifact correction; *ICA*: independent component analysis, *low-pass*: low-pass filter in Hertz; *high-pass*: high-pass filter in Hertz; *baseline*: baseline interval in milliseconds; *autoreject* version either interpolate (*interp*) or reject artifact-contaminated trials (*reject*).

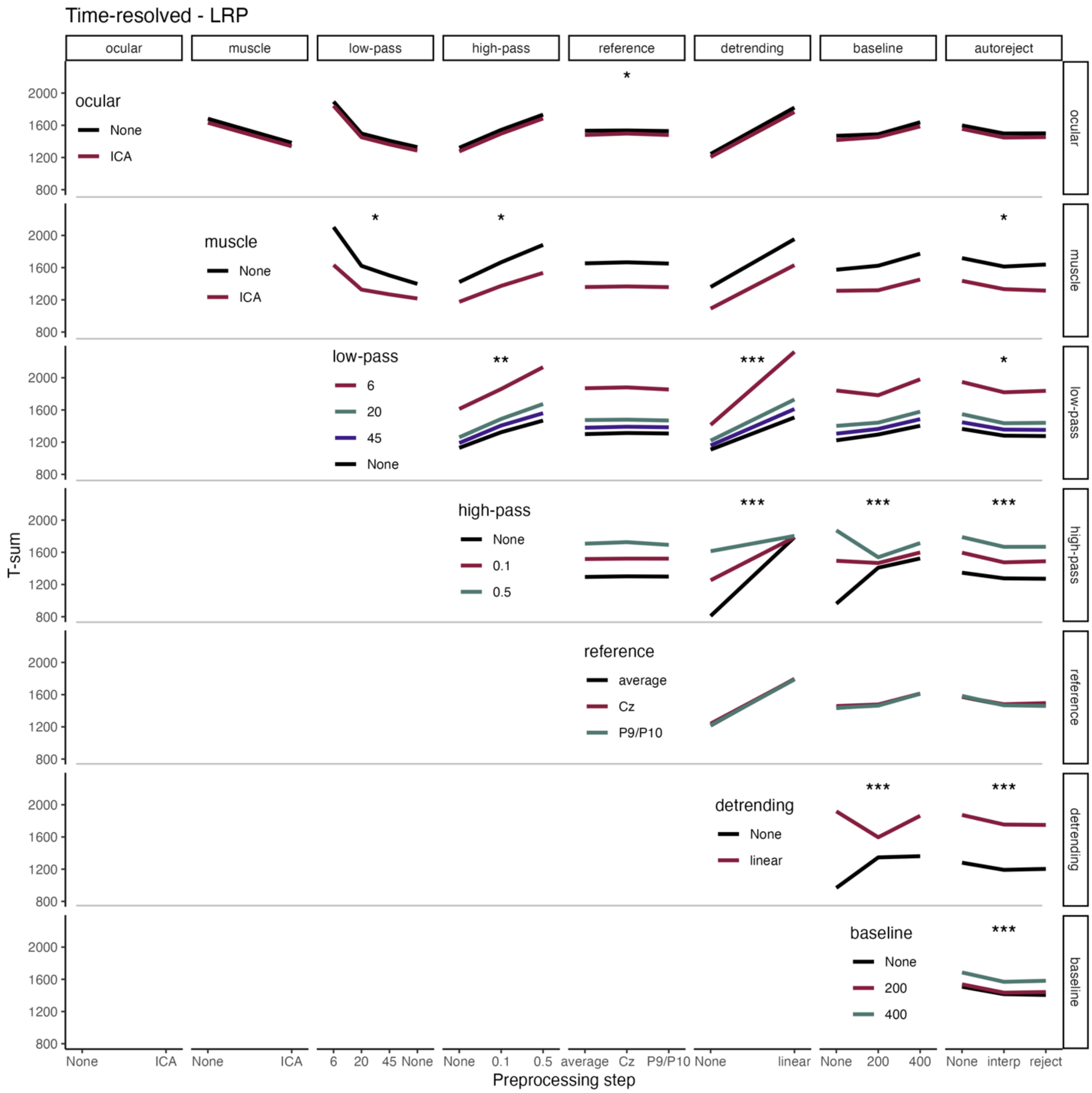

Fig. S13. Interactions between preprocessing steps on decoding performance for LRP and time-resolved decoding.
See Figure S12 for details.

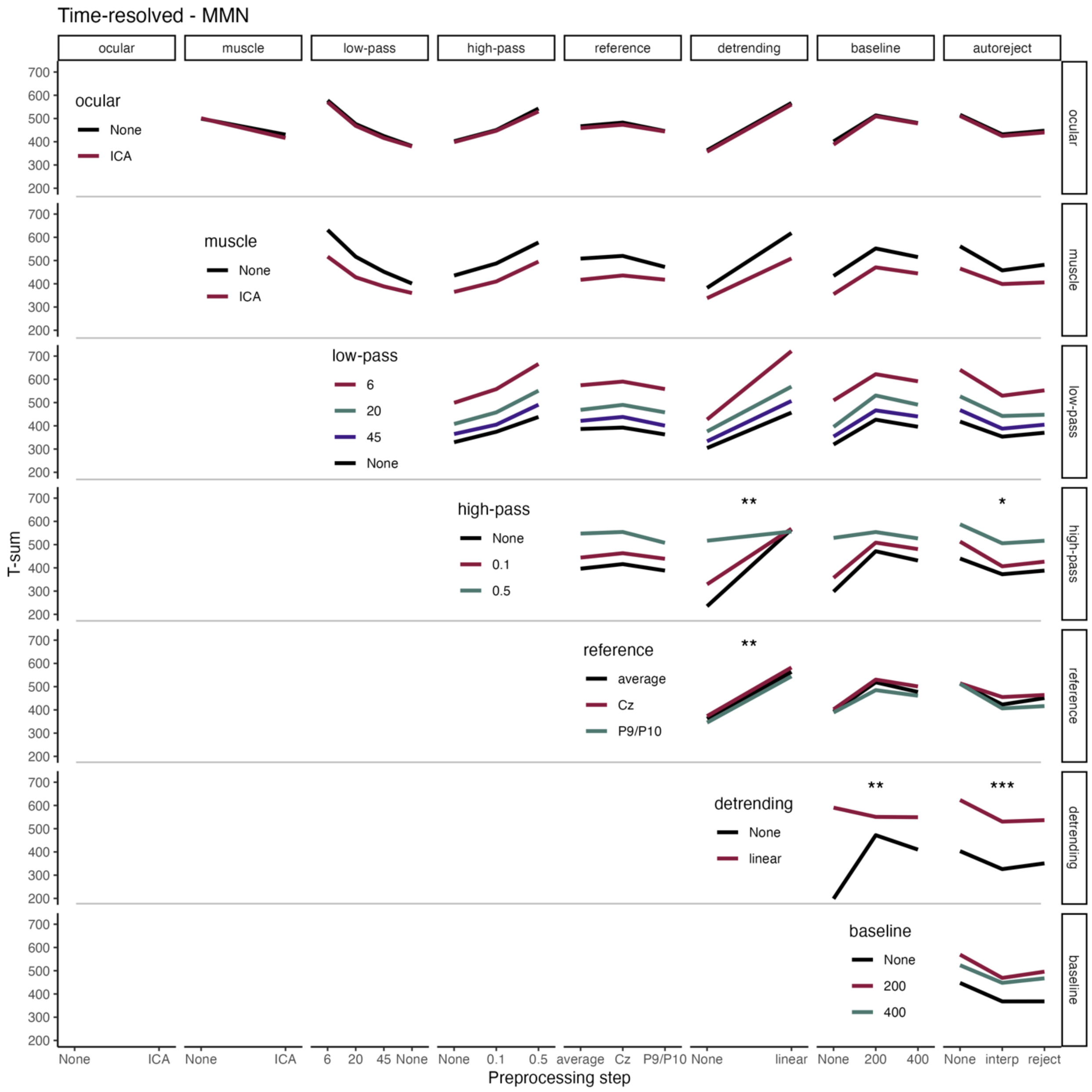

Fig. S14. Interactions between preprocessing steps on decoding performance for MMN and time-resolved decoding.
See Figure S12 for details.

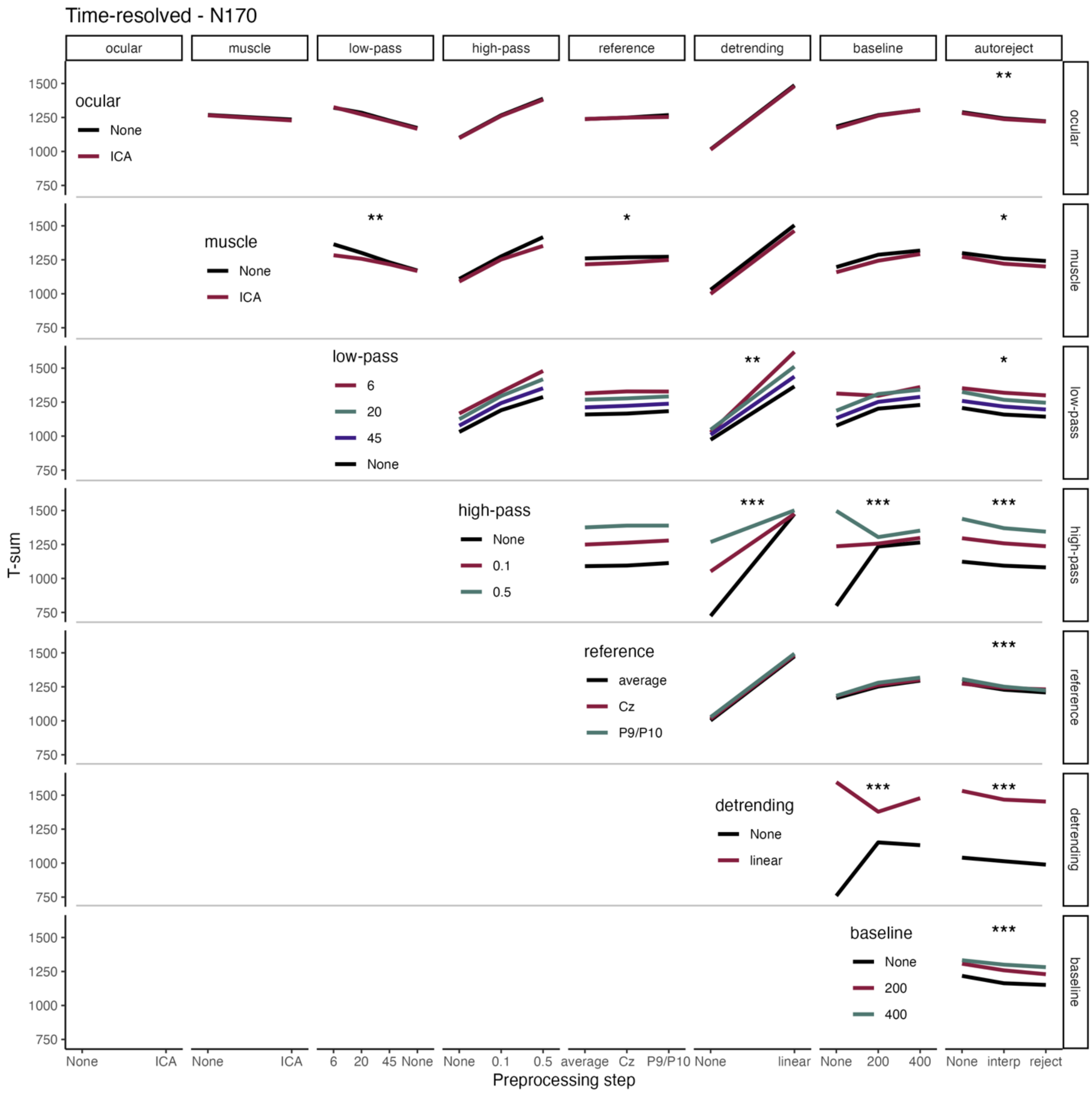

Fig. S15. Interactions between preprocessing steps on decoding performance for N170 and time-resolved decoding.

See Figure S12 for details.

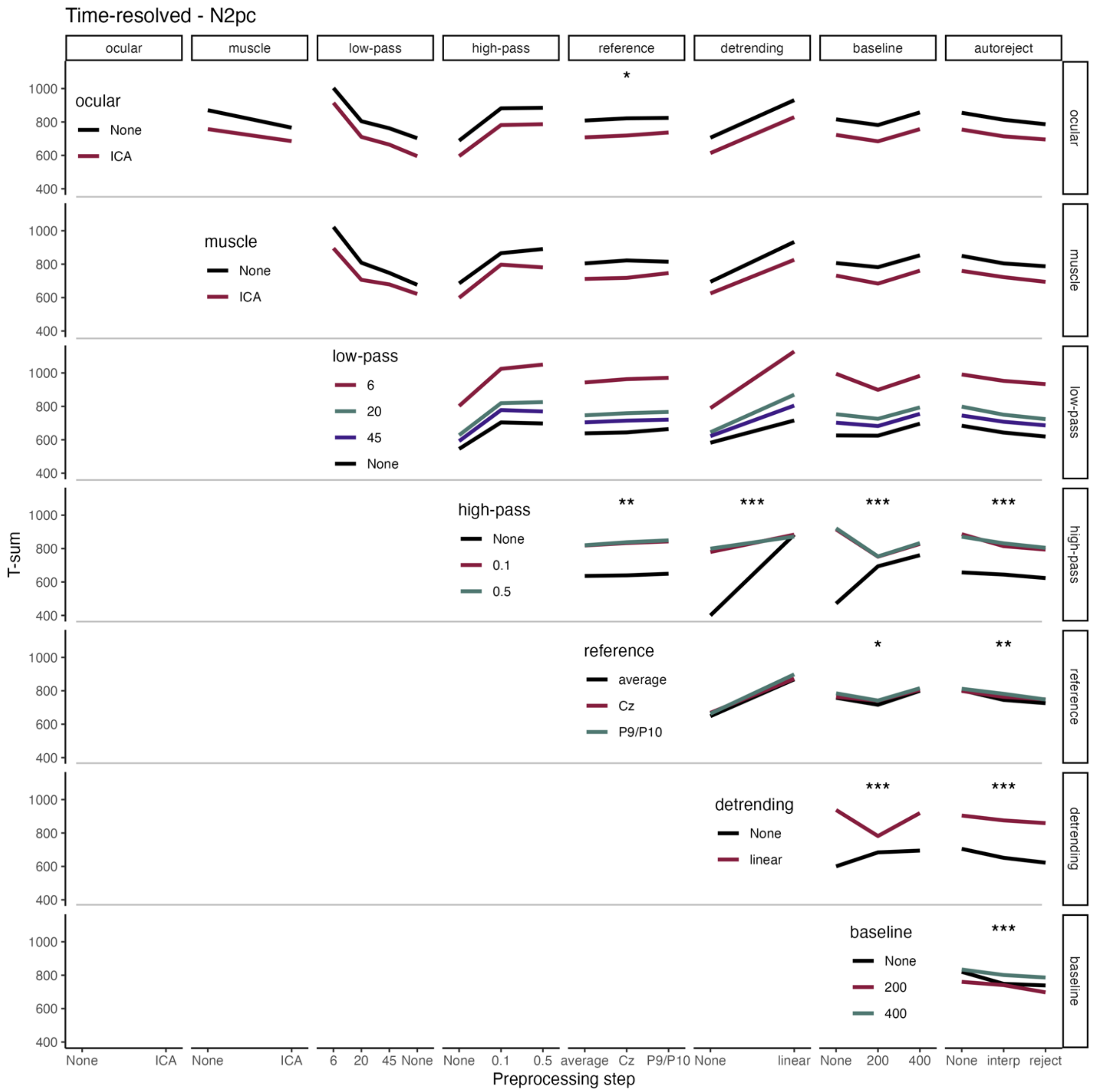

Fig. S16. Interactions between preprocessing steps on decoding performance for N2pc and time-resolved decoding.
See Figure S12 for details.

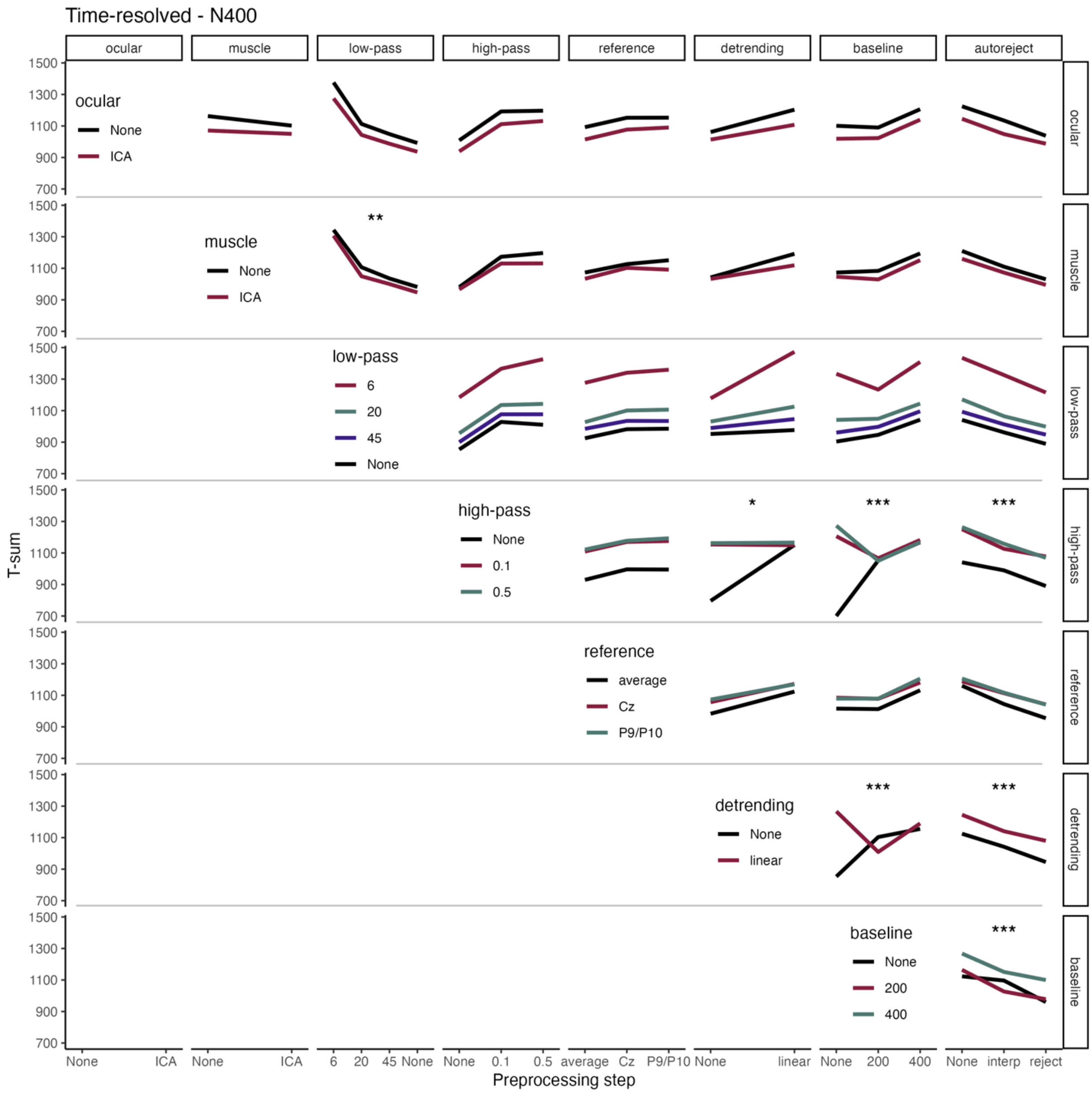

Fig. S17. Interactions between preprocessing steps on decoding performance for N400 and time-resolved decoding.
See Figure S12 for details.

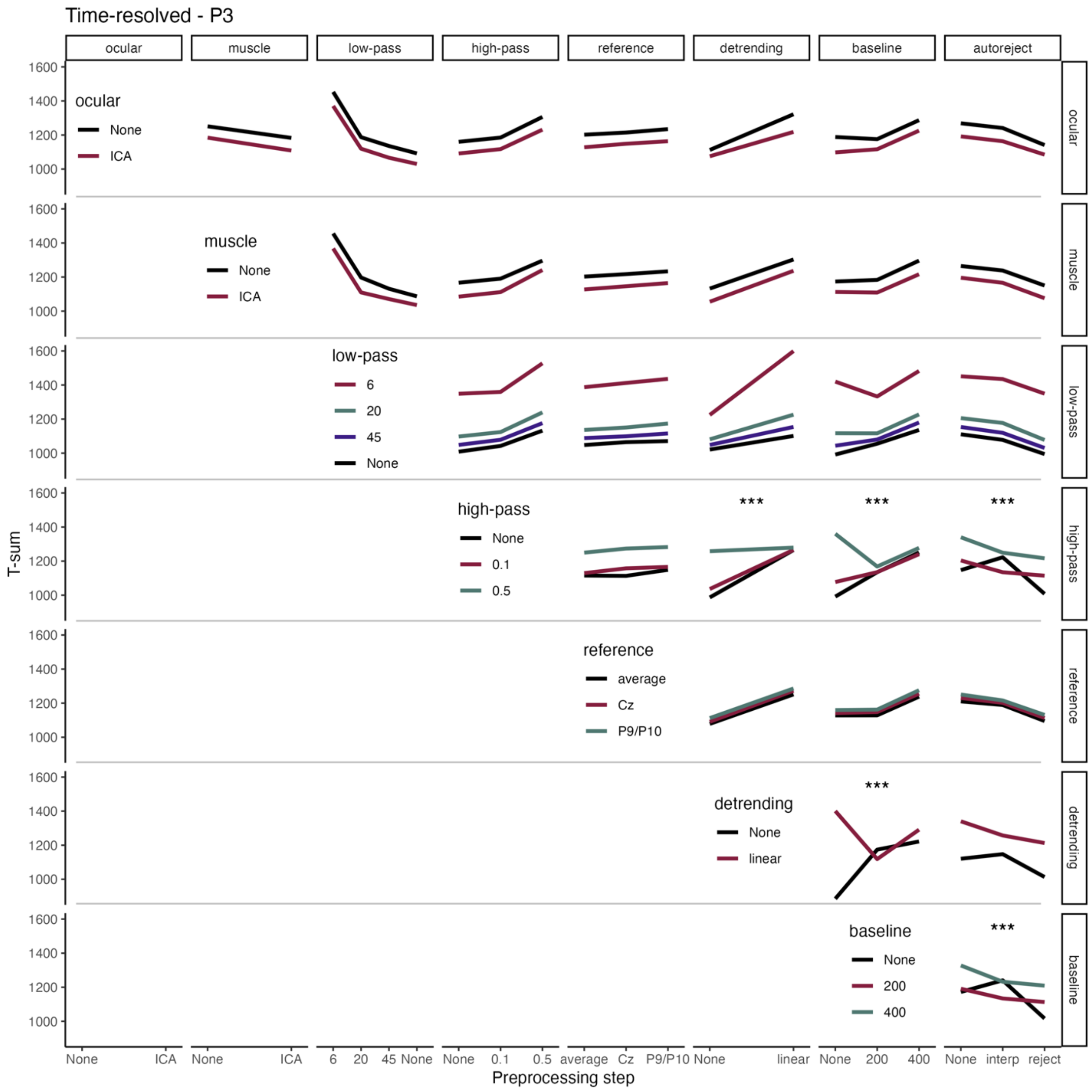

Fig. S18. Interactions between preprocessing steps on decoding performance for P3 and time-resolved decoding.
See Figure S12 for details.

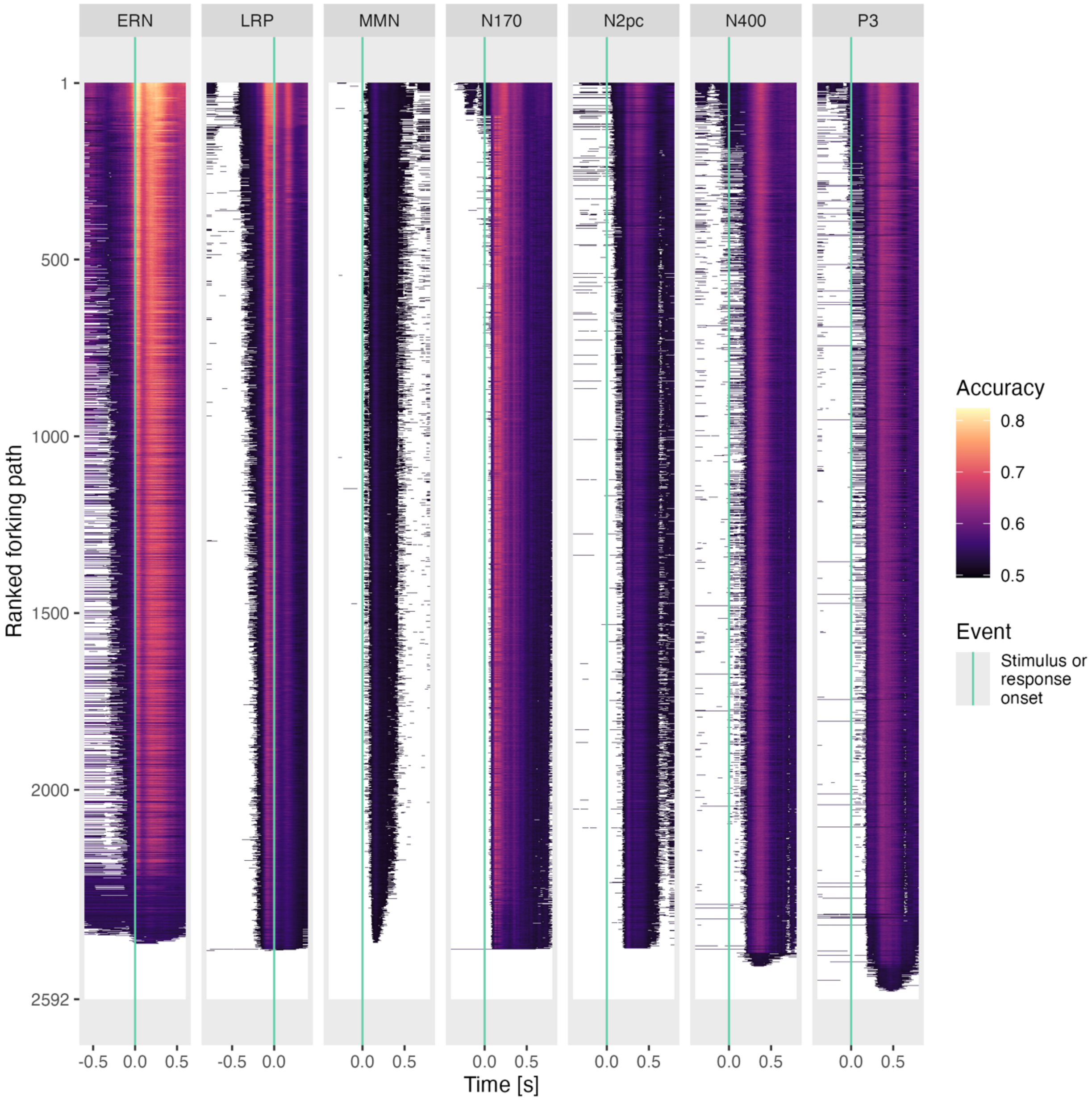

Fig. S19. Time-resolved decoding accuracy for all forking paths.
The decoding accuracy for each time point (x-axes) is color-coded. The forking paths of each experiment (horizontal panels) are ranked based on their *T*-sum from #1 (best) to #2592 (worst) on the y-axes, and therefore correspond to the forking paths of Figure S5. The vertical aquamarine line illustrates stimulus or response onset (Table S5). Only time points are color-coded, which fall in a significant cluster as defined by cluster-permutation testing.

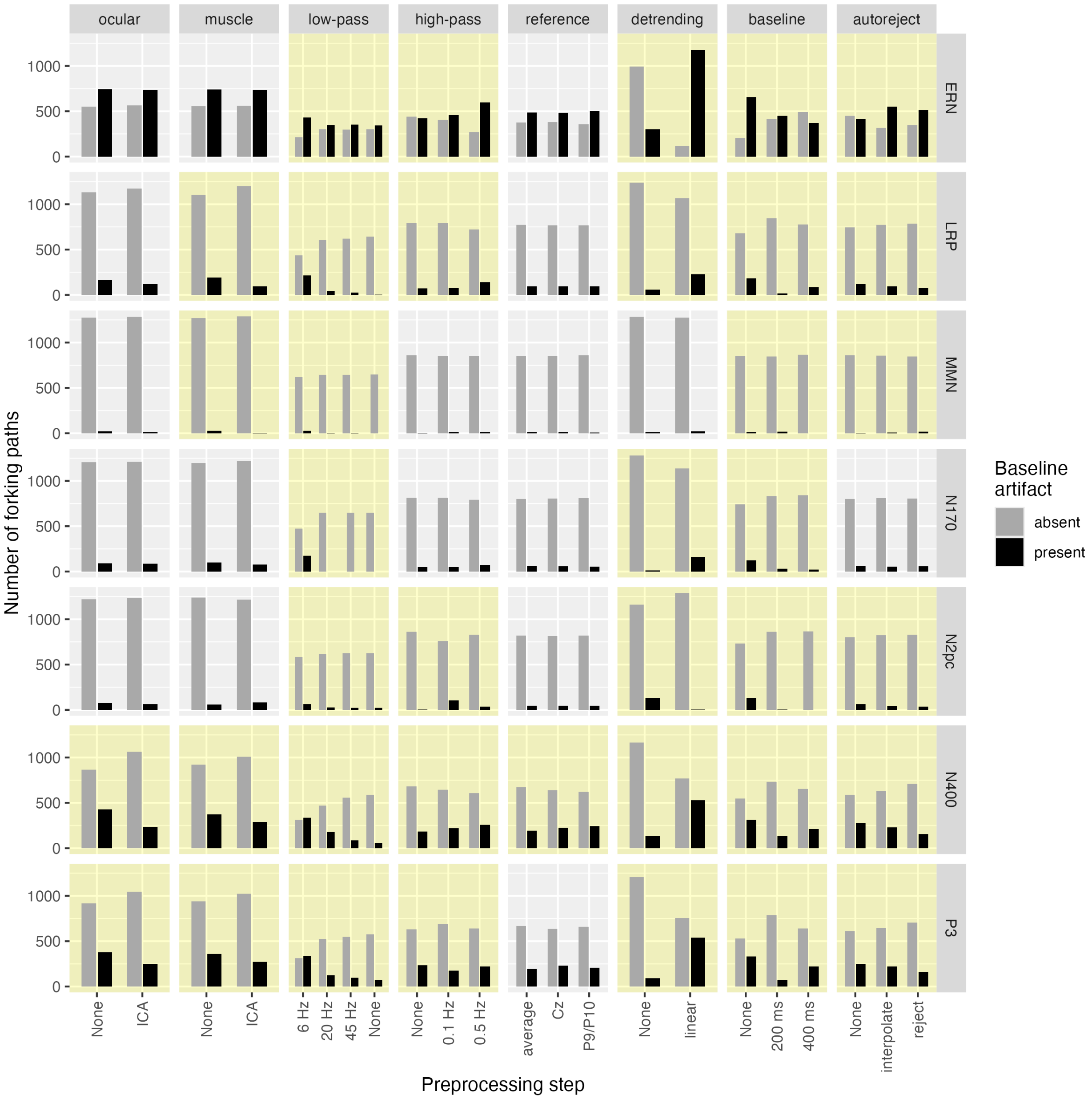

Fig. S20. Influence of preprocessing steps on the presence or absence of a baseline artifact in time-resolved decoding.

For each experiment (vertical panels) and preprocessing step (horizontal panels) and its respective version (x-axes), the number of forking paths producing a so-called baseline artifact during time-resolved decoding is counted (y-axes). Grey bars illustrate the number of forking paths without such an artifact, and black bars the number of forking paths with such an artifact. Yellow facets indicate significant likelihood ratio tests ($p < 0.01$) of a processing step within an experiment.

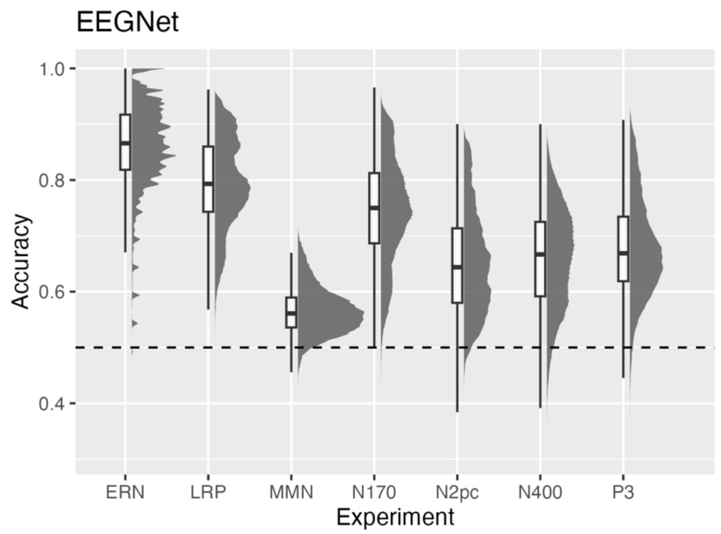

Fig. S21. Overview of EEGNet decoding accuracies.
(Balanced) Decoding accuracies (y-axis) are illustrated for each forking path and participant, separately for each experiment (x-axis). Contrary to Figure 3A, no averaging across participants was performed for each forking path. One can observe the higher variability in decoding accuracy when not removing the participant variability compared to Figure 3A. Boxes represent the interquartile range (25th to 75th percentile), with the median indicated by a solid black line. Whiskers extend to the most extreme values within 1.5 times the interquartile range from the lower and upper quartiles.

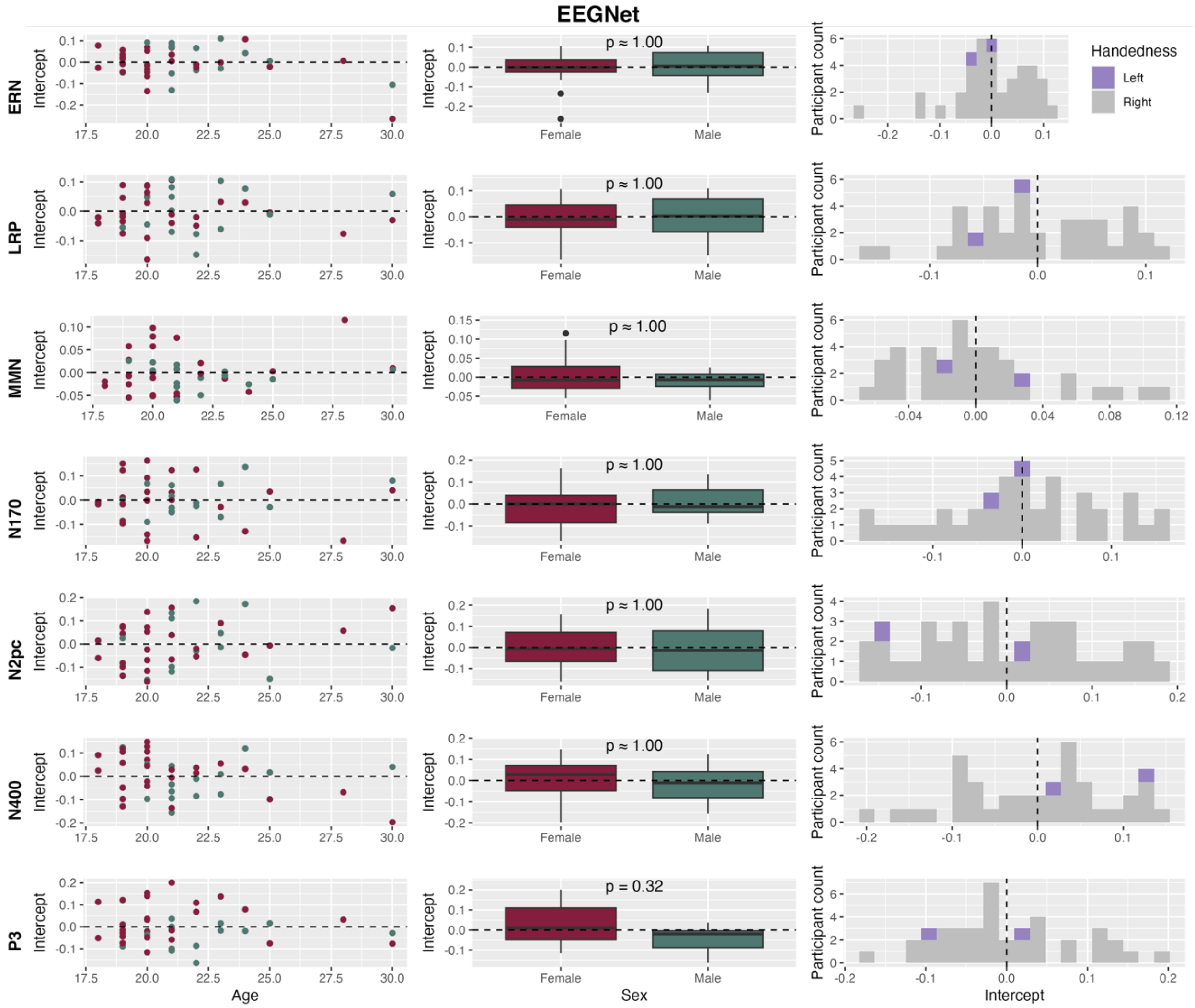

Fig. S22. Relation between random intercepts and participant demographics using EEGNet.

For each experiment (row), each participant's random intercept – i.e., the individual offset in decoding accuracy – are plotted against demographic variables (see Supplementary Text – Influence of the participant in the LMMs). Left column: The relationship between age and random intercept, grouped by sex (red = female, green = male). Middle column: The association between sex and random intercept. *P*-values represent the results of a false discovery rate-corrected Man-Whitney *U* test using the Benjamini Hochberg adjustment. No association was significant. Boxes represent the interquartile range (25th to 75th percentile), with the median indicated by a solid black line. Whiskers extend to the most extreme values within 1.5 times the interquartile range from the lower and upper quartiles. Data points beyond this range are shown as outliers. Right column: Histogram of the relationship between random intercept and handedness. Only two participants in the dataset were left-handed.

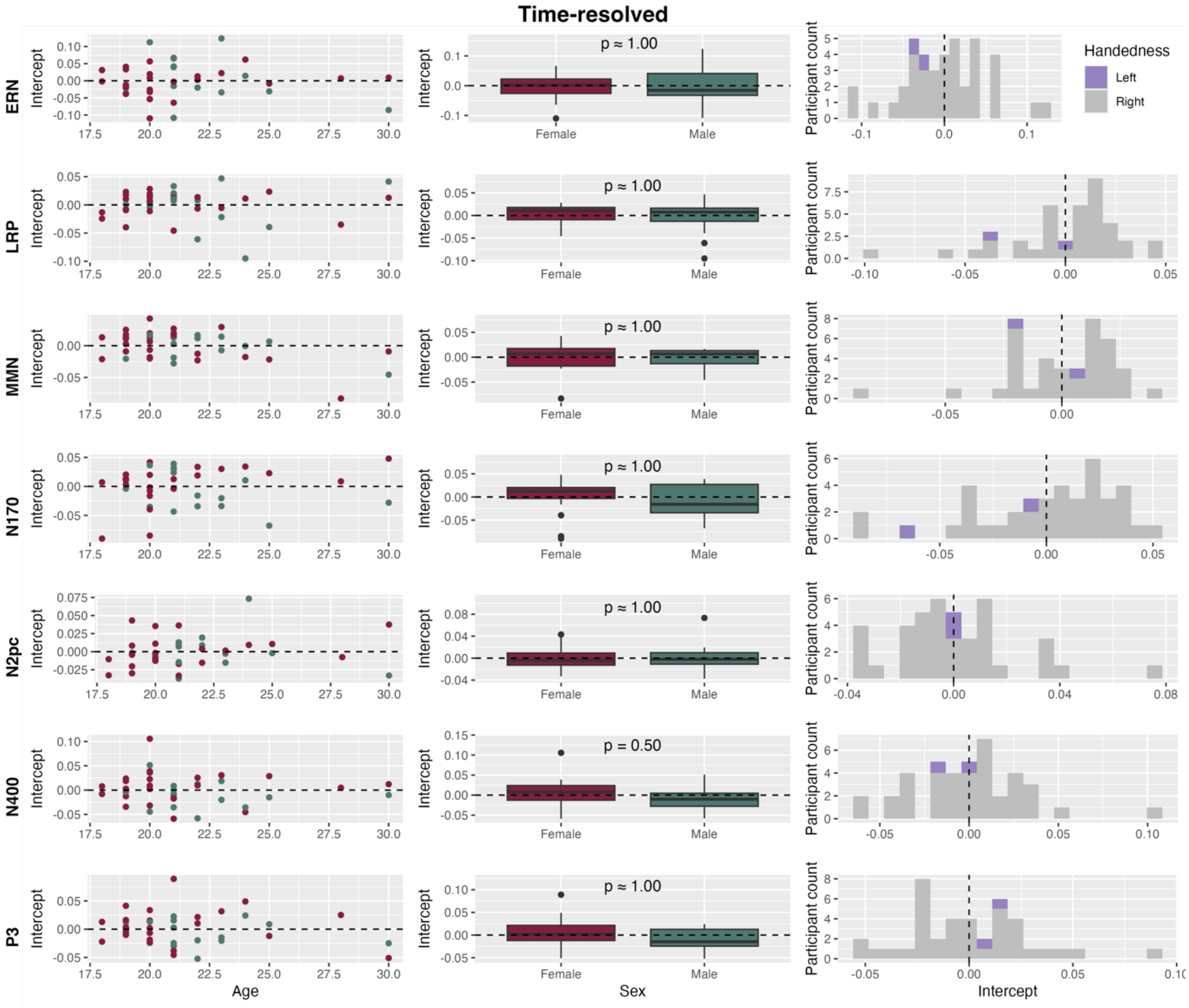

Fig. S23. Relation between random intercepts and participant demographics using time-resolved decoding.
See Figure S22 for details. No association was significant.

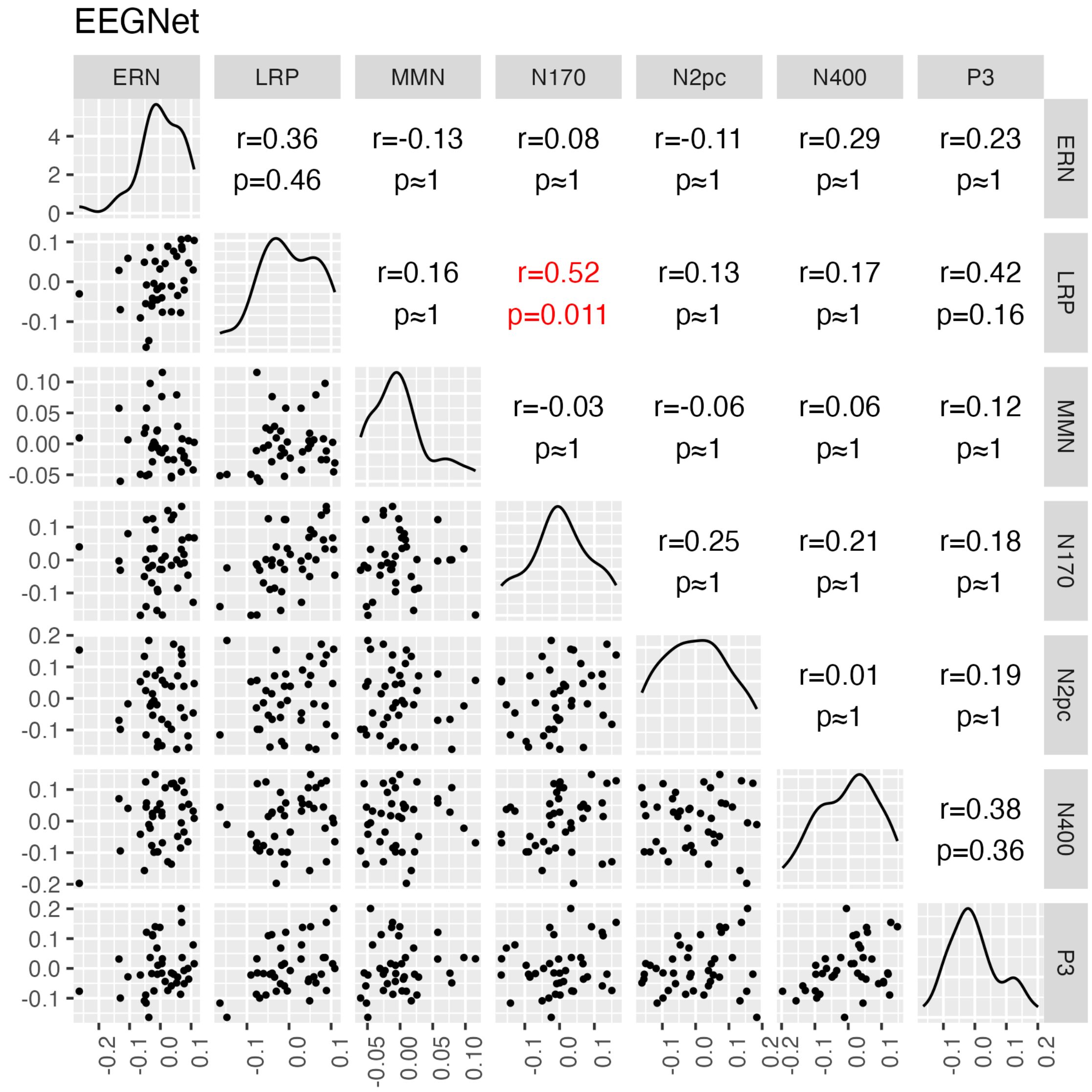

Fig. S24. Correlations of random intercepts between experiments (EEGNet decoding). Pairwise Pearson correlations were computed, correlating the random intercepts of the same participants but in two different experiments and therefore extracted from two different LMMs (see Supplementary Text – Influence of the participant in the LMMs). The lower diagonal illustrates the random intercepts of the same participants but across two experiments and LMMs, extracted from these LMMs. The on-diagonal elements show a kernel density estimate of the distributions. The upper diagonal illustrates the Pearson correlation coefficients and associated false discovery rate-corrected *p*-values.

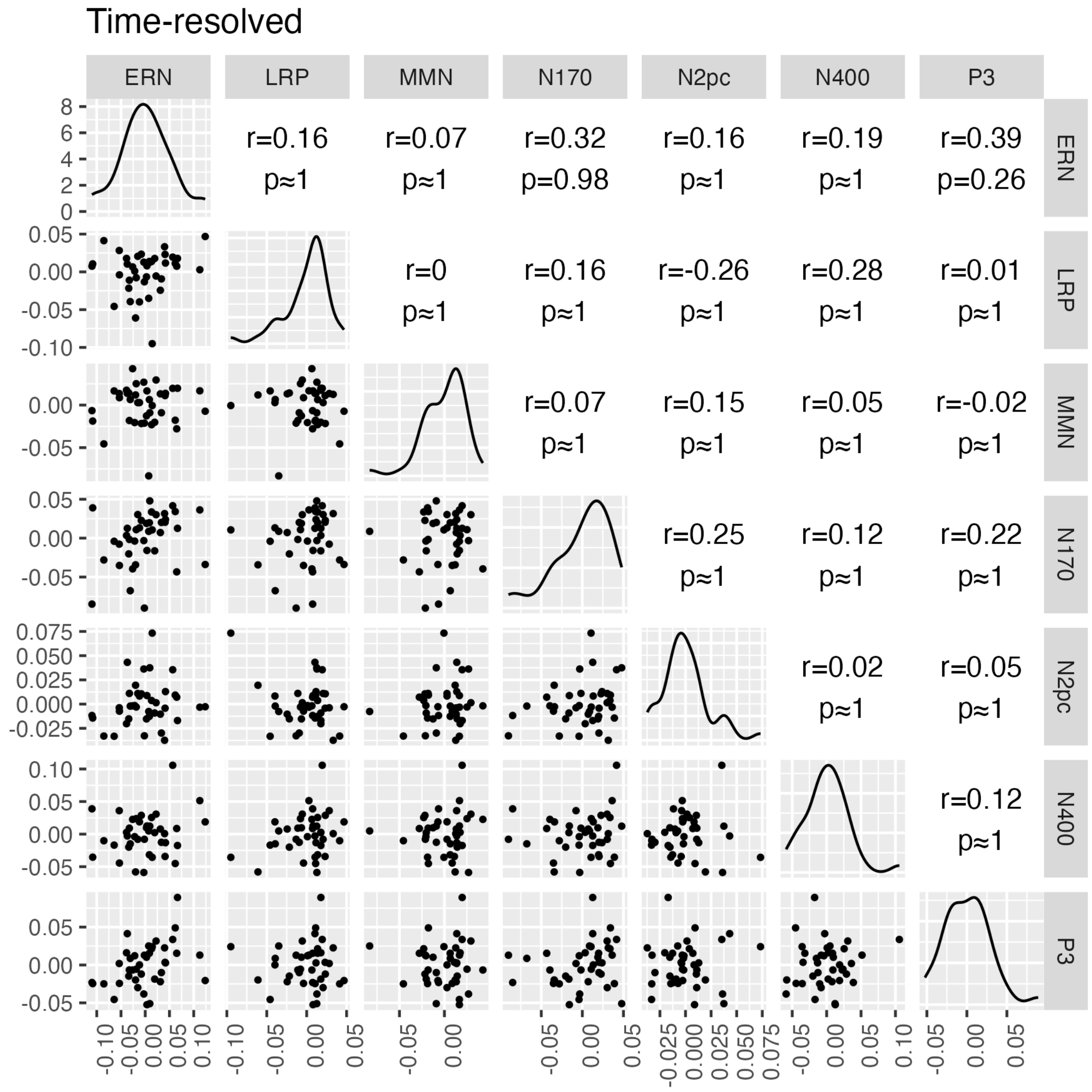

Fig. S25. Correlations of random intercepts between experiments (time-resolved decoding). See Figure S24 for details.

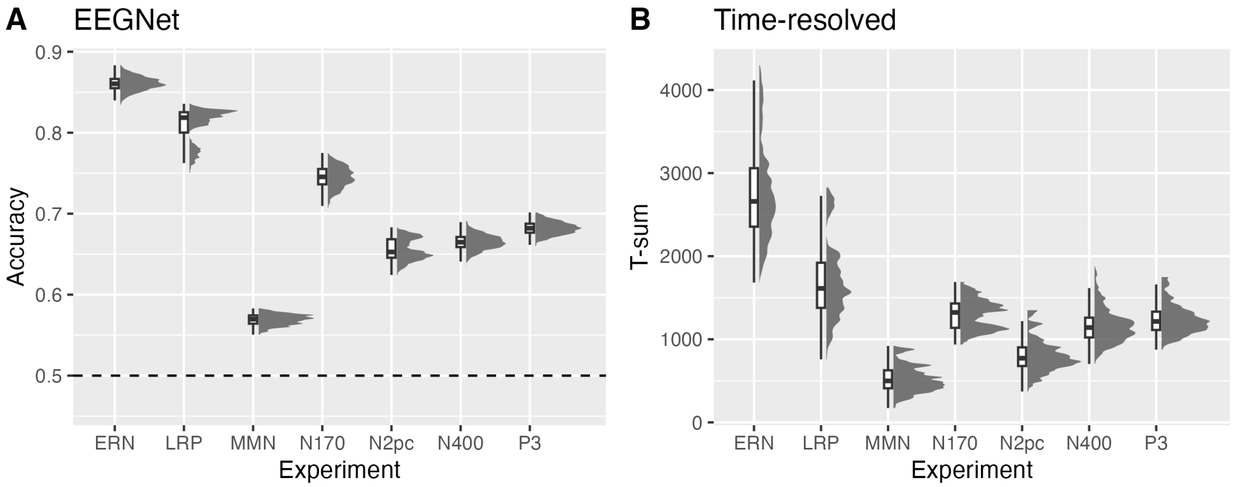

Fig. S26. Overview of decoding performances in an alternative multiverse.
The alternative multiverse comprised a different order of preprocessing steps, i.e. (1) re-referencing, (2) HPF & LPF, (3) ocular artifact correction, (4) muscle artifact correction, (5) baseline correction & detrending, and (6) autoreject (see Supplementary Text – Alternative order of preprocessing steps). **A**: (Balanced) Decoding accuracies (y-axis) are plotted for each forking path (averaged across participants), separately for each experiment (x-axis). **B**: *T*-sums (y-axis) are plotted for each forking path and across participants, separately for each experiment (x-axis). Boxes represent the interquartile range (25th to 75th percentile), with the median indicated by a solid black line. Whiskers extend to the most extreme values within 1.5 times the interquartile range from the lower and upper quartiles.

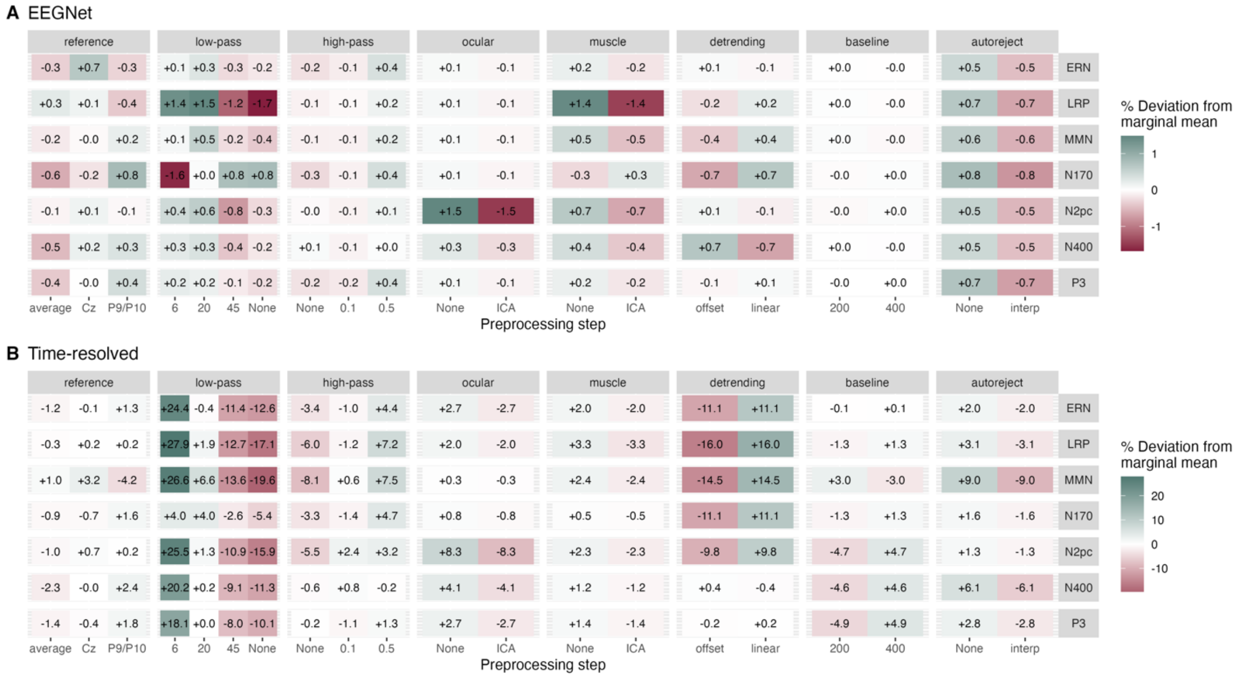

Fig. S27. Influence of preprocessing steps on decoding performance in an alternative multiverse.

The alternative multiverse comprised a different order of preprocessing steps, i.e. (1) re-referencing, (2) HPF & LPF, (3) ocular artifact correction, (4) muscle artifact correction, (5) baseline correction & detrending, and (6) autoreject (see Supplementary Text – Alternative order of preprocessing steps). Percentage deviation from marginal means of either decoding accuracy (EEGNet, **A**) or *T*-sum (time-resolved, **B**) are depicted within each tile. Marginal means for each level (x-axis) of preprocessing step (horizontal panels) are normalized to the mean of the respective experiment (vertical panels), and shows the percentage differences in relation to this mean value, and not the absolute improvement or decline in decoding accuracy as a percentage. Color scales differ in **A** and **B**. All cells are colored. The horizontal panels are ordered according to the preprocessing steps in the pipeline. The order differs with respect to Figure 5. See Figure 5 for details.

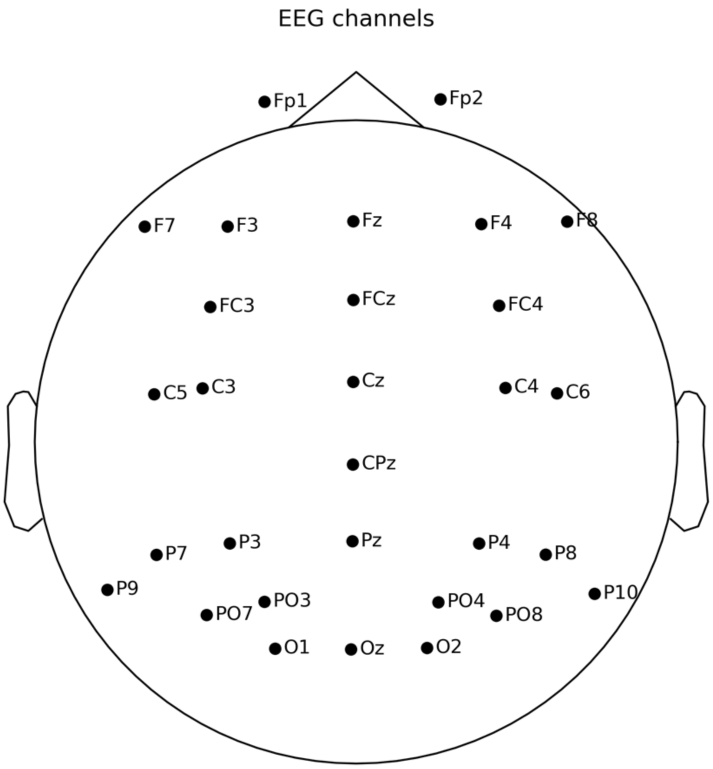

Fig. S28. Schematic of the EEG montage.
30 scalp electrodes were recorded along with three EOG electrodes (not shown).

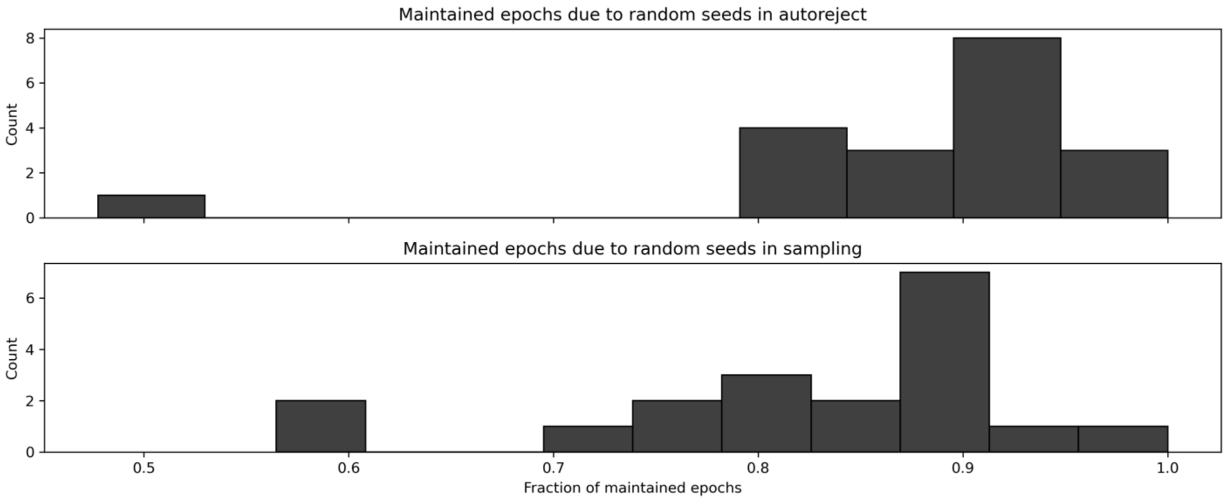

Fig. S29. Fraction of maintained epochs for different seeds.
Autoreject was deployed using one example participant of the ERN experiment (see Supplementary Text – Variability due to random seed in autoreject). The fraction of maintained epochs (x-axes) for different random seeds was counted across seeds (y-axes). Upper panel: Seed varied in autoreject function. Lower panel: Seed varied in epoch sampling.

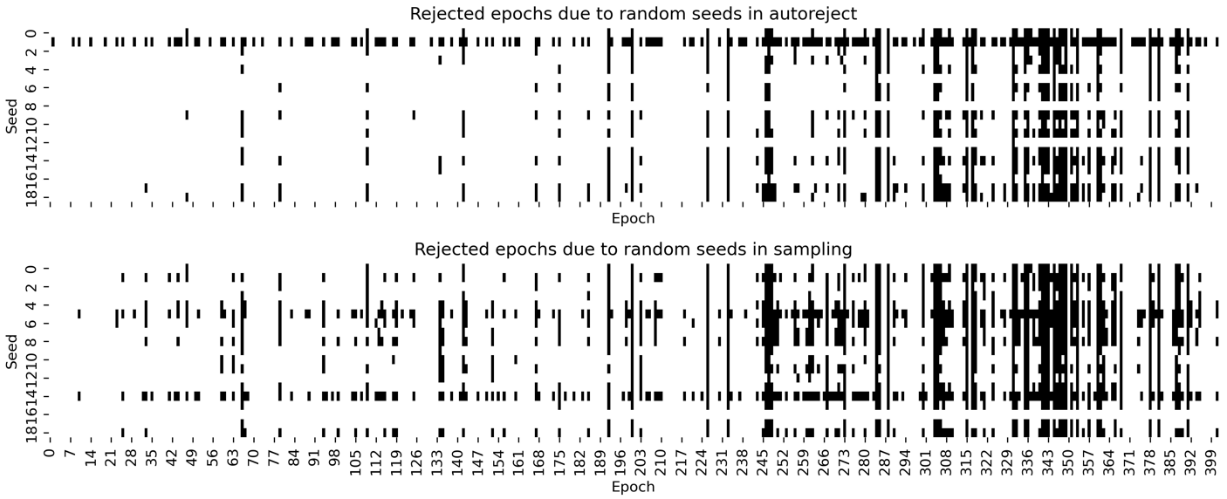

Fig. S30. Rejected epochs for different seeds.
Autoreject was deployed using one example participant of the ERN experiment (see Supplementary Text – Variability due to random seed in autoreject). For each epoch (x-axes) and random seed (y-axes), black tiles indicate rejection of an epoch due to exceeding the estimated threshold, whereas white tiles indicate epochs which were maintained. Upper panel: Seed varied in autoreject function. Lower panel: Seed varied in epoch sampling.

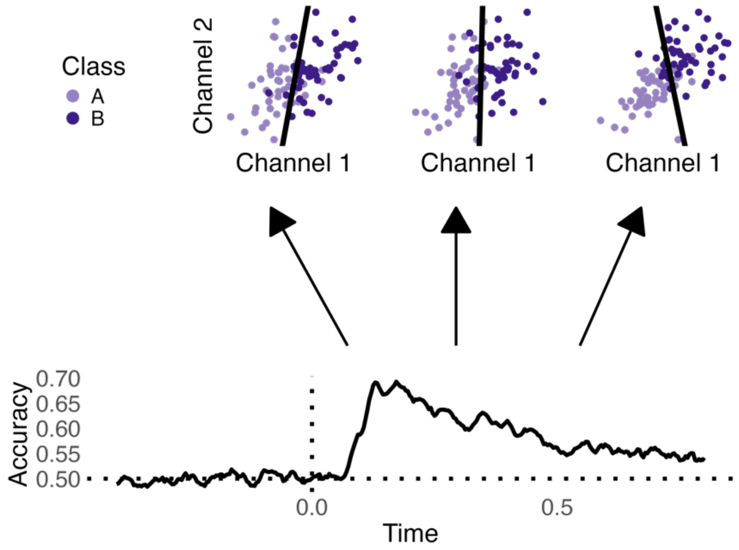

Fig. S31. Schematic of time-resolved decoding.

In time-resolved decoding analysis, all channel values (here, electrode voltages) from all trials at each respective time point are used to train and test binary classifiers (top panel). In the present study, logistic regressions were used. The (cross-validated, balanced) test accuracies are then written into a decoding time series (lower panel), which is further analyzed across participants.

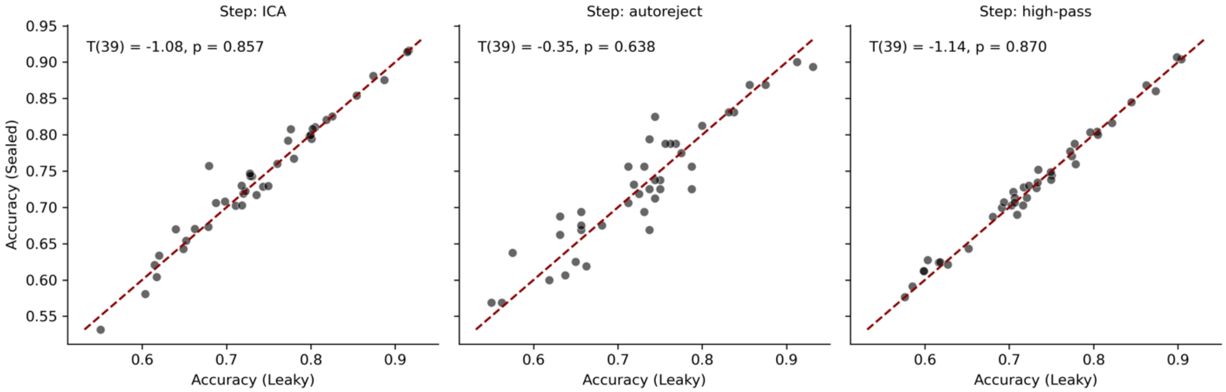

Figure S32: The influence of latent leakage on decoding performance. For the N170 experiment and some example forking paths, we preprocessed the data using a sealed and a leaky version of some processing steps, i.e., ICA, autoreject, and HPF (see Supplementary Text – Influence of latent leakage on decoding performance). We fitted an EEGNet classifier for each participant and pipeline. Balanced accuracy estimates are plotted for the sealed (y-axes) versus the leaky (x-axes) versions and each processing step (horizontal facets). The dashed red line indicates the identity line. One-tailed paired-sampled *T*-tests did not indicate higher accuracies for leaky pipelines (all $p > 0.638$).

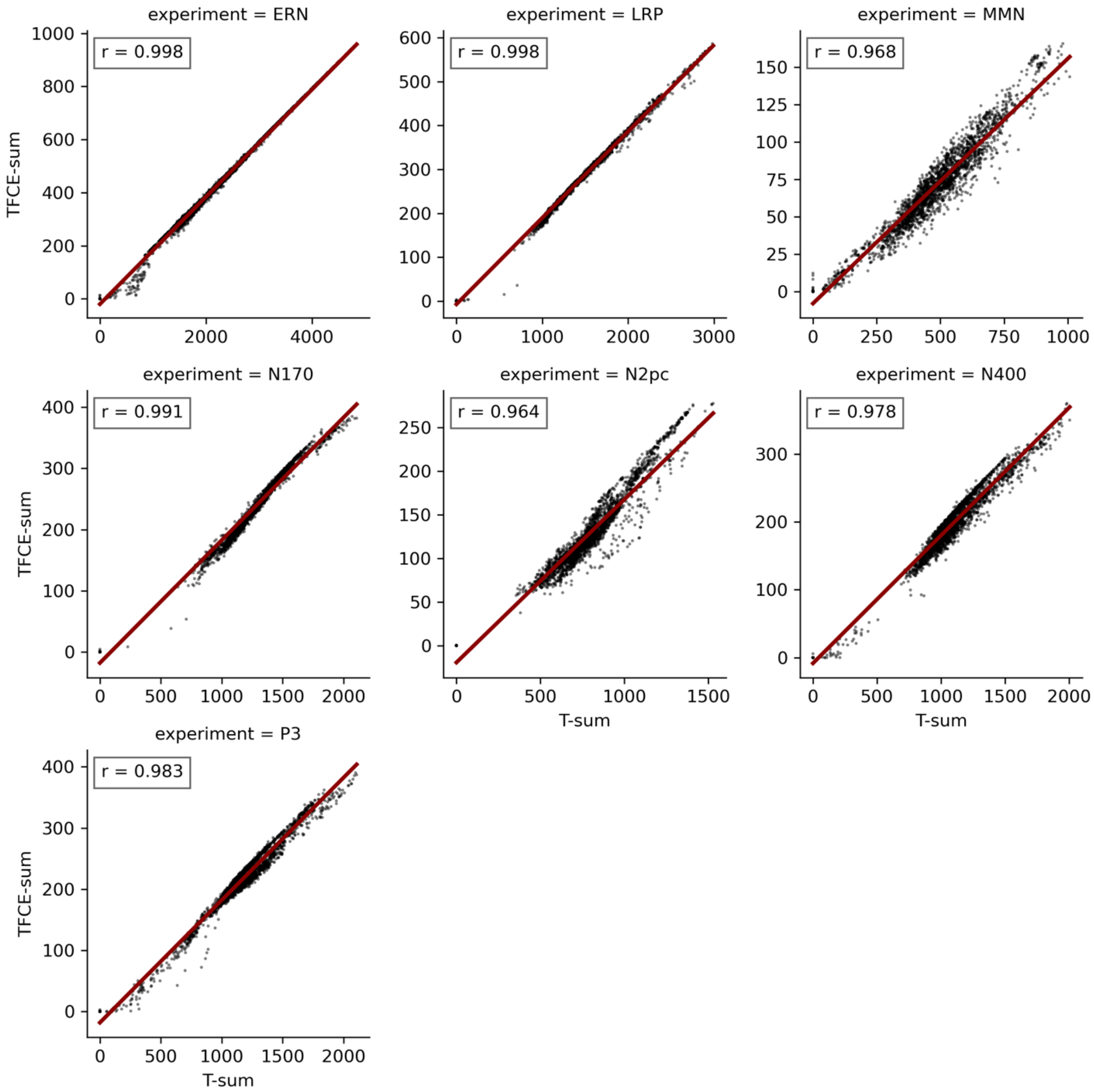

Figure S33: Correlation between *TFCE*-sum and *T*-sum per experiment. We first used an arbitrary cluster-forming threshold (corresponding to $p < 0.05$) and secondly *TFCE* (hyperparameters: start = 0, step = 0.2), and quantified the *T*-sums (x-axes) or *TFCE*-sums (y-axes), separately for each forking path and experiment across participants. Red lines represent linear fits to the data. Both measures were strongly correlated across forking paths (Pearson correlation, all $r > 0.96$).

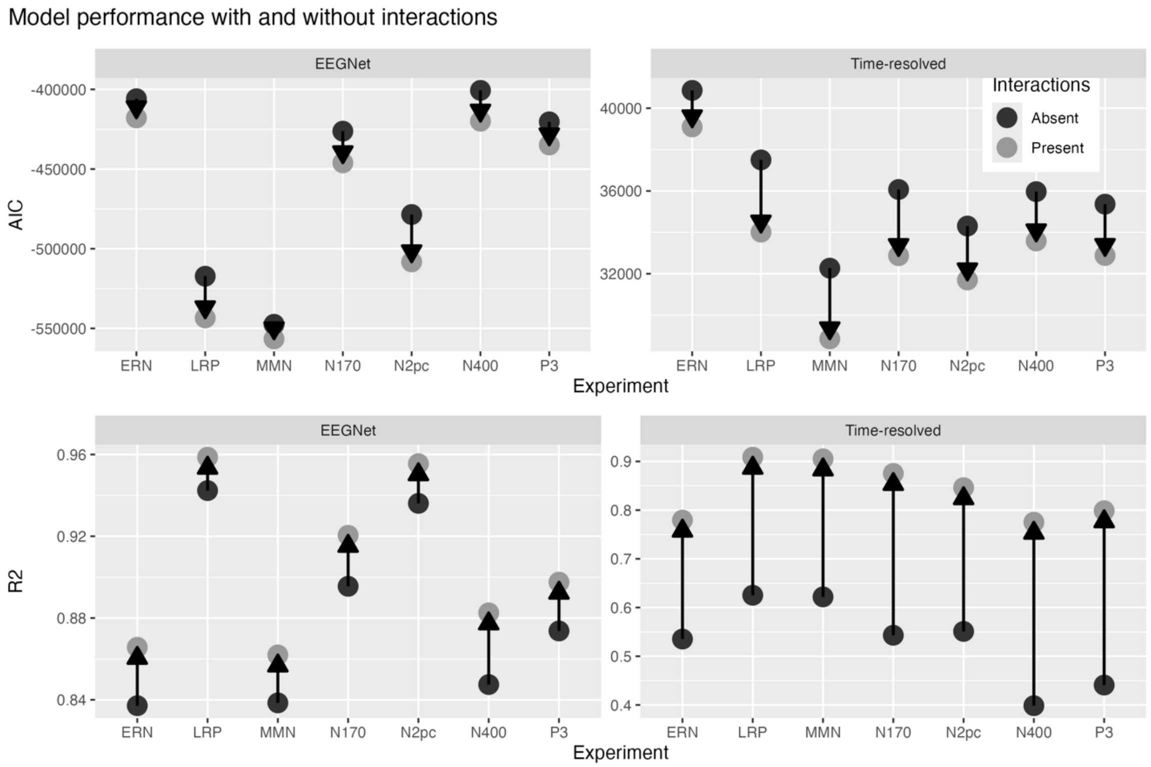

Fig. S34. AIC and R^2 values for models without interactions and models with two-way interactions included.

AIC values (upper facets) and R^2 values (lower panels) are illustrated for models without interactions (black dots) or with two-way interactions (gray dots). Models are LMMs for EEGNet (left panels) and LMs for time-resolved decoding (right panels). Arrows indicate the change from models without interactions towards models with interactions. Including interaction terms consistently decreased AIC values, supporting the decision to include these terms in our model. The R^2 values from EEGNet compared to time-resolved models were already high without interaction values (note different scales of y-axes), indicating that a large fraction of variance was already explained by the main effects.

Table S1.

Significant effects of preprocessing on EEGNet decoding performance, separately for each experiment. *F*-tests were performed for each processing step. Stars indicate the significance ('.' $p < 0.1$; '*' $p < 0.05$; '**' $p < 0.01$; '***' $p < 0.001$). *Ocular*: ocular artifact correction; *muscle*: muscle artifact correction; *ICA*: independent component analysis, *low-pass*: low-pass filter; *high-pass*: high-pass filter.

**model term**	**ERN**	**LRP**	**MMN**	**N170**	**N2pc**	**N400**	**P3**
ocular					*		
muscle		***	**		*	.	
low-pass	.			***			
high-pass	***	***		**	*		
reference	**	.	.	*		**	
detrending		**	**	***			
baseline	*	***		***	***	***	***
autoreject	***	***	**	***	**	.	***
ocular:muscle	.						
ocular:low-pass							
ocular:high-pass			.				
ocular:reference		*			*		
ocular:detrending					.		
ocular:baseline							
ocular:autoreject				**			
muscle:low-pass	**	*		**		**	
muscle:high-pass		*					
muscle:reference				*			
muscle:detrending	.		.				
muscle:baseline		.					
muscle:autoreject		*		*			.
low-pass:high-pass		**					
low-pass:reference	*			.			
low-pass:detrending	.	***		**			.
low-pass:baseline							
low-pass:autoreject		*		*			.
high-pass:reference					**		
high-pass:detrending	*	***	**	***	***	*	***
high-pass:baseline		***	.	***	***	***	***
high-pass:autoreject	***	***	*	***	***	***	***
reference:detrending			**				
reference:baseline					*		
reference:autoreject		.		***	**		
detrending:baseline	***	***	**	***	***	***	***
detrending:autoreject		***	***	***	***	***	.
baseline:autoreject	*	***	.	***	***	***	***

Table S2.

Significant effects of preprocessing on time-resolved decoding performance, separately for each experiment. See Table S1 for details.

model term	ERN	LRP	MMN	N170	N2pc	N400	P3
ocular	***	***	**		***	***	***
muscle	***	***	***	***	***	***	***
low-pass	***	***	***	***	***	***	***
high-pass	***	***	***	***	***	***	***
reference	.		***	**	***	***	***
detrending	***	***	***	***	***	***	***
baseline	***	***	***	***	***	***	***
autoreject	***	***	***	***	***	***	***
ocular:muscle	*		***		***	**	
ocular:low-pass						*	
ocular:high-pass							
ocular:reference							
ocular:detrending	*					***	***
ocular:baseline	.						*
ocular:autoreject						*	
muscle:low-pass	*	***	***	***	***		*
muscle:high-pass	***	***		***	***	**	.
muscle:reference			***		**	.	
muscle:detrending		***	***		***	***	
muscle:baseline		***			.		
muscle:autoreject		**	***				
low-pass:high-pass	***	***	***	*	***	**	***
low-pass:reference							
low-pass:detrending	***	***	***	***	***	***	***
low-pass:baseline	***	***	*	***	***	***	***
low-pass:autoreject			***			**	
high-pass:reference			***				
high-pass:detrending	***	***	***	***	***	***	***
high-pass:baseline	**	***	***	***	***	***	***
high-pass:autoreject	***	*	***	*	***	***	***
reference:detrending					*	*	
reference:baseline			***				
reference:autoreject			***		.	*	
detrending:baseline	***	***	***	***	***	***	***
detrending:autoreject	***	*	***	*	**	.	***
baseline:autoreject			***		***	***	***

Table S3.

For each experiment, pairwise post-hoc comparisons in EEGNet decoding performance within each preprocessing step using Tukey adjustment. See Table S1 for details.

variable	level 1	level 2	ERN	LRP	MMN	N170	N2pc	N400	P3
ocular	None	ICA					*		
muscle	None	ICA		***	**		*	.	
low-pass	None	6 Hz	.			***			
low-pass	None	20 Hz							
low-pass	None	45 Hz							
low-pass	6 Hz	20 Hz	*			***			
low-pass	6 Hz	45 Hz	*			***			
low-pass	20 Hz	45 Hz							
high-pass	None	0.1 Hz	.	**		*			
high-pass	None	0.5 Hz	**	***	.	*			
high-pass	0.1 Hz	0.5 Hz							
reference	average	Cz	**					*	
reference	average	P9/P10		*	.	*		*	
reference	Cz	P9/P10	*						
detrending	None	linear		**	**	***			
baseline	None	200 ms		***		**	***	*	**
baseline	None	400 ms		***		***	***	**	***
baseline	200 ms	400 ms							
autoreject	None	interpolate	**	***	*	***	.		***
autoreject	None	reject	**	***		**	*		*
autoreject	interpolate	reject							

Table S4.

For each experiment, pairwise post-hoc comparisons in time-resolved decoding performance within each preprocessing step using Tukey adjustment. See Table S1 for details.

**variable**	**level 1**	**level 2**	**ERN**	**LRP**	**MMN**	**N170**	**N2pc**	**N400**	**P3**
ocular	None	ICA	***	***	**		***	***	***
muscle	None	ICA	***	***	***	***	***	***	***
low-pass	None	6 Hz	***	***	***	***	***	***	***
low-pass	None	20 Hz	***	***	***	***	***	***	***
low-pass	None	45 Hz	**	***	***	***	***	***	***
low-pass	6 Hz	20 Hz	***	***	***	***	***	***	***
low-pass	6 Hz	45 Hz	***	***	***	***	***	***	***
low-pass	20 Hz	45 Hz	**	***	***	***	***	***	***
high-pass	None	0.1 Hz	***	***	***	***	***	***	***
high-pass	None	0.5 Hz	***	***	***	***	***	***	***
high-pass	0.1 Hz	0.5 Hz	***	***	***	***			***
reference	average	Cz			***		.	***	*
reference	average	P9/P10	.		***	**	***	***	***
reference	Cz	P9/P10			***				*
detrending	None	linear	***	***	***	***	***	***	***
baseline	None	200 ms	***	**	***	***	***		
baseline	None	400 ms	***	***	***	***	***	***	***
baseline	200 ms	400 ms		***	***	***	***	***	***
autoreject	None	interpolate	***	***	***	***	***	***	***
autoreject	None	reject		***	***	***	***	***	***
autoreject	interpolate	reject	***		***	**	***	***	***

Table S5.

Time-locking events, epoch intervals, and baseline reference and intervals. The total duration of the epochs is kept equal across experiments. The baseline interval varied per forking path, either not applied, or a window of 200 ms or 400 ms. The illustrated baseline intervals are with respect to the baseline reference. The illustrated epoch intervals are with respect to the time-locking event. The length of the entire trials and the post-baseline time windows was kept equal between forking paths.

**experiment/ component**	**time-locking event**	**epoch interval [ms]**	**baseline reference**	**baseline interval [ms]**	
				200 ms	400 ms
ERN	response	(-600; 600)	stimulus	(-200; 0)	(-400; 0)
LRP	response	(-800; 400)	response	(-600; -400)	(-800; -400)
MMN	stimulus	(-400; 800)	stimulus	(-200; 0)	(-400; 0)
N170	stimulus	(-400; 800)	stimulus	(-200; 0)	(-400; 0)
N2pc	stimulus	(-400; 800)	stimulus	(-200; 0)	(-400; 0)
N400	stimulus	(-400; 800)	stimulus	(-200; 0)	(-400; 0)
P3	stimulus	(-400; 800)	stimulus	(-200; 0)	(-400; 0)

Table S6.
High-, low-, and band-pass filter characteristics during multiverse preprocessing. All filters used a Hamming window, a passband ripple of 0.0194 and a stopband attenuation of 53 dB. The filters were automatically determined by *MNE* based on low- and high-pass cutoffs in the respective forking path (first two columns). The bottom row shows the filter applied to the raw data prior to ICA.

Low-pass (Hz)	High-pass (Hz)	Filter type	Transition bandwidth (Hz)	-6 dB Cutoff frequency (Hz)	Filter length (samples)	Filter duration (s)
None	0.1	high-pass	0.1	0.05	8449	33.004
None	0.5	high-pass	0.5	0.25	1691	6.605
6	None	low-pass	2	7	423	1.652
6	0.1	band-pass	0.10 / 2.00	0.05 / 7.00	8449	33.004
6	0.5	band-pass	0.50 / 2.00	0.25 / 7.00	1691	6.605
30	None	low-pass	7.5	33.75	113	0.441
30	0.1	band-pass	0.10 / 7.50	0.05 / 33.75	8449	33.004
30	0.5	band-pass	0.50 / 7.50	0.25 / 33.75	1691	6.605
45	None	low-pass	11.25	50.62	77	0.301
45	0.1	band-pass	0.10 / 11.25	0.05 / 50.62	8449	33.004
45	0.5	band-pass	0.50 / 11.25	0.25 / 50.62	1691	6.605
**Filter preceding ICA:**						
None	1.0	high-pass	1.0	0.5	845	3.301

Table S7.
EEGNet hyperparameters.

name	value	default or manual
n_chans / in_chans	30	manual
n_outputs / n_classes	2	manual
n_times / input_window_samples	308	manual
final_conv_length	'auto'	
pool_mode	'mean'	
F1	8	default
D	2	default
F2	16	default
depthwise_kernel_length	16	default
pool1_kernel_size	4	default
pool2_kernel_size	8	default
kernel_length	64	default
conv_spatial_max_norm	1	default
activation	ELU	default
batch_norm_momentum	0.01	default
batch_norm_affine	True	default
batch_norm_eps	0.001	default
final_layer_with_constraint	False	default
norm_rate	0.25	default
drop_prob	0.25	
input_window_seconds	None	
sfreq	256	manual
max_epochs	200	manual
batch_size	16	manual
lr (learning rate)	0.01	default
optimizer	torch.optim.SGD	default
criterion	torch.nn.NLLLoss	default

Table S8.
EEGNet architecture. EEGNet was deployed for trials with length 1.2 s (308 samples) and 30 channels.

Layer	Input Shape	Output Shape	Param #	Kernel Shape
EEGNetv4 (EEGNetv4)	[1, 30, 308]	[1, 2]	--	--
├─ Ensure4d (ensuredims): 1-1	[1, 30, 308]	[1, 30, 308, 1]	--	--
├─ Rearrange (dimshuffle): 1-2	[1, 30, 308, 1]	[1, 1, 30, 308]	--	--
├─ Conv2d (conv_temporal): 1-3	[1, 1, 30, 308]	[1, 8, 30, 309]	512	[1, 64]
├─ BatchNorm2d (bnorm_temporal): 1-4	[1, 8, 30, 309]	[1, 8, 30, 309]	16	--
├─ Conv2dWithConstraint (conv_spatial): 1-5	[1, 8, 30, 309]	[1, 16, 1, 309]	480	[30, 1]
├─ BatchNorm2d (bnorm_1): 1-6	[1, 16, 1, 309]	[1, 16, 1, 309]	32	--
├─ Expression (elu_1): 1-7	[1, 16, 1, 309]	[1, 16, 1, 309]	--	--
├─ AvgPool2d (pool_1): 1-8	[1, 16, 1, 309]	[1, 16, 1, 77]	--	[1, 4]
├─ Dropout (drop_1): 1-9	[1, 16, 1, 77]	[1, 16, 1, 77]	--	--
├─ Conv2d (conv_separable_depth): 1-10	[1, 16, 1, 77]	[1, 16, 1, 78]	256	[1, 16]
├─ Conv2d (conv_separable_point): 1-11	[1, 16, 1, 78]	[1, 16, 1, 78]	256	[1, 1]
├─ BatchNorm2d (bnorm_2): 1-12	[1, 16, 1, 78]	[1, 16, 1, 78]	32	--
├─ Expression (elu_2): 1-13	[1, 16, 1, 78]	[1, 16, 1, 78]	--	--
├─ AvgPool2d (pool_2): 1-14	[1, 16, 1, 78]	[1, 16, 1, 9]	--	[1, 8]
├─ Dropout (drop_2): 1-15	[1, 16, 1, 9]	[1, 16, 1, 9]	--	--
├─ Sequential (final_layer): 1-16	[1, 16, 1, 9]	[1, 2]	--	--
│ └─Conv2d (conv_classifier): 2-1	[1, 16, 1, 9]	[1, 2, 1, 1]	290	[1, 9]
│ └─Rearrange (permute_back): 2-2	[1, 2, 1, 1]	[1, 2, 1, 1]	--	--
│ └─Expression (squeeze): 2-3	[1, 2, 1, 1]	[1, 2]	--	--

Table S9.
Time-resolved logistic regression hyperparameters.

name	value	default or manual
C	1.0	default
class_weight	balanced	manual
penalty	L2	default
dual	False	default
tolerance	0.0001	default
solver	liblinear	manual
dual	False	default
fit_intercept	True	default
intercept_scaling	1	default
l1_ratio	None	default
max_iter	100	default
multi_class	auto	default
warm_start	False	default

References

1. Jas, M., Engemann, D. A., Bekhti, Y., Raimondo, F. & Gramfort, A. Autoreject: Automated artifact rejection for MEG and EEG data. *NeuroImage* **159**, 417–429 (2017).
2. Jas, M., Engemann, D., Raimondo, F., Bekhti, Y. & Gramfort, A. Automated rejection and repair of bad trials in MEG/EEG. in *6th International Workshop on Pattern Recognition in Neuroimaging (PRNI)* (Trento, Italy, 2016).
3. Shrout, P. E. & Fleiss, J. L. Intraclass correlations: uses in assessing rater reliability. *Psychol. Bull.* **86**, 420 (1979).
4. Cicchetti, D. V. Guidelines, criteria, and rules of thumb for evaluating normed and standardized assessment instruments in psychology. *Psychol. Assess.* **6**, 284 (1994).
5. Winkler, I., Debener, S., Muller, K.-R. & Tangermann, M. On the influence of high-pass filtering on ICA-based artifact reduction in EEG-ERP. in *2015 37th Annual International Conference of the IEEE Engineering in Medicine and Biology Society (EMBC)* 4101–4105 (IEEE, Milan, 2015). doi:10.1109/EMBC.2015.7319296.
6. Benjamini, Y. & Hochberg, Y. Controlling the false discovery rate: a practical and powerful approach to multiple testing. *J. R. Stat. Soc. Ser. B Methodol.* **57**, 289–300 (1995).
7. Ehinger, B. V. & Bonasch, H. Decoding accuracies as well as ERP amplitudes do not show between-task correlations. Preprint at (2023).
