# Supplementary material for: How EEG preprocessing shapes decoding performance
Source: Commun Biol. 2025 Jul 10;8:1039. doi: 10.1038/s42003-025-08464-3 (PMC12246244; doi:10.1038/s42003-025-08464-3)
Supplement: Supplementary file 3 — Reporting summary [file 42003_2025_8464_MOESM3_ESM.pdf]

nature portfolio

Corresponding author(s): Roman Kessler

Last updated by author(s): May 15, 2025

Reporting Summary

Nature Portfolio wishes to improve the reproducibility of the work that we publish. This form provides structure for consistency and transparency in reporting. For further information on Nature Portfolio policies, see our Editorial Policies and the Editorial Policy Checklist.

Statistics

For all statistical analyses, confirm that the following items are present in the figure legend, table legend, main text, or Methods section.

n/a	Confirmed	
☐	☒	The exact sample size (*n*) for each experimental group/condition, given as a discrete number and unit of measurement
☐	☒	A statement on whether measurements were taken from distinct samples or whether the same sample was measured repeatedly
☐	☒	The statistical test(s) used AND whether they are one- or two-sided<br>*Only common tests should be described solely by name; describe more complex techniques in the Methods section.*
☐	☒	A description of all covariates tested
☐	☒	A description of any assumptions or corrections, such as tests of normality and adjustment for multiple comparisons
☐	☒	A full description of the statistical parameters including central tendency (e.g. means) or other basic estimates (e.g. regression coefficient) AND variation (e.g. standard deviation) or associated estimates of uncertainty (e.g. confidence intervals)
☐	☒	For null hypothesis testing, the test statistic (e.g. *F*, *t*, *r*) with confidence intervals, effect sizes, degrees of freedom and *P* value noted<br>*Give P values as exact values whenever suitable.*
☒	☐	For Bayesian analysis, information on the choice of priors and Markov chain Monte Carlo settings
☒	☐	For hierarchical and complex designs, identification of the appropriate level for tests and full reporting of outcomes
☒	☐	Estimates of effect sizes (e.g. Cohen's *d*, Pearson's *r*), indicating how they were calculated

Our web collection on statistics for biologists contains articles on many of the points above.

Software and code

Policy information about availability of computer code

Data collection	A GitHub repository containing all scripts is available at https://github.com/kesslerr/m4d. The raw dataset used in our analyses is shared by its original authors at https://osf.io/thsqg/.
Data analysis	A GitHub repository containing all scripts is available at https://github.com/kesslerr/m4d. The raw dataset used in our analyses is shared by its original authors at https://osf.io/thsqg/.

For manuscripts utilizing custom algorithms or software that are central to the research but not yet described in published literature, software must be made available to editors and reviewers. We strongly encourage code deposition in a community repository (e.g. GitHub). See the Nature Portfolio guidelines for submitting code & software for further information.

Data

Policy information about availability of data

All manuscripts must include a data availability statement. This statement should provide the following information, where applicable:

- Accession codes, unique identifiers, or web links for publicly available datasets
- A description of any restrictions on data availability
- For clinical datasets or third party data, please ensure that the statement adheres to our policy

A GitHub repository containing all scripts is available at https://github.com/kesslerr/m4d, including Bash & Python scripts handling the complete multiverse preprocessing and decoding on a high-performance computing cluster. The repository also comprises all scripts to compute LMMs in Julia, and to perform all

remaining analyses in R based on a targets pipeline. Comprehensive lists of package versions in all used programming languages are available at https://github.com/kesslerr/m4d/tree/main/env.

Research involving human participants, their data, or biological material

Policy information about studies with human participants or human data. See also policy information about sex, gender (identity/presentation), and sexual orientation and race, ethnicity and racism.

Reporting on sex and gender	We used an openly available dataset, which was described by their original authors in more detail: Kappenman, E. S., Farrens, J. L., Zhang, W., Stewart, A. X. & Luck, S. J. ERP CORE: An open resource for human event-related potential research. NeuroImage 225 117465 (2021).
Reporting on race, ethnicity, or other socially relevant groupings	see above
Population characteristics	see above
Recruitment	see above
Ethics oversight	see above

Note that full information on the approval of the study protocol must also be provided in the manuscript.

Field-specific reporting

Please select the one below that is the best fit for your research. If you are not sure, read the appropriate sections before making your selection.

☒ Life sciences ☐ Behavioural & social sciences ☐ Ecological, evolutionary & environmental sciences

For a reference copy of the document with all sections, see nature.com/documents/nr-reporting-summary-flat.pdf

Life sciences study design

All studies must disclose on these points even when the disclosure is negative.

Sample size	We used the entire public dataset published earlier (see above). No sample size was determined.
Data exclusions	No participants were discarded. Single trials were excluded in single forking paths as described in the methods section based on the estimated threshold from the autoreject package.
Replication	All scripts and the original data are shared by us or the original datasets authors. The targets pipeline is designed for exact reproducibility of the R analyses.
Randomization	We used all participants for each analysis. No grouping or randomization was performed.
Blinding	Not relevant because no grouping.

Reporting for specific materials, systems and methods

We require information from authors about some types of materials, experimental systems and methods used in many studies. Here, indicate whether each material, system or method listed is relevant to your study. If you are not sure if a list item applies to your research, read the appropriate section before selecting a response.

Materials & experimental systems

n/a	Involved in the study	
☒	☐	Antibodies
☒	☐	Eukaryotic cell lines
☒	☐	Palaeontology and archaeology
☒	☐	Animals and other organisms
☒	☐	Clinical data
☒	☐	Dual use research of concern
☒	☐	Plants

Methods

n/a	Involved in the study	
☒	☐	ChIP-seq
☒	☐	Flow cytometry
☒	☐	MRI-based neuroimaging

Plants

Seed stocks	*Report on the source of all seed stocks or other plant material used. If applicable, state the seed stock centre and catalogue number. If plant specimens were collected from the field, describe the collection location, date and sampling procedures.*
Novel plant genotypes	*Describe the methods by which all novel plant genotypes were produced. This includes those generated by transgenic approaches, gene editing, chemical/radiation-based mutagenesis and hybridization. For transgenic lines, describe the transformation method, the number of independent lines analyzed and the generation upon which experiments were performed. For gene-edited lines, describe the editor used, the endogenous sequence targeted for editing, the targeting guide RNA sequence (if applicable) and how the editor was applied.*
Authentication	*Describe any authentication procedures for each seed stock used or novel genotype generated. Describe any experiments used to assess the effect of a mutation and, where applicable, how potential secondary effects (e.g. second site T-DNA insertions, mosiacism, off-target gene editing) were examined.*
